# Mesenchymal Stem Cells Ameliorate Acute‐on‐Chronic Liver Failure Associated With Expansion of Immunoregulatory Hepatic NKp46^+^DX5^−^ NK Cells

**DOI:** 10.1155/jimr/2373331

**Published:** 2026-09-24

**Authors:** Jing Xiong, Weizhen Weng, Dengna Lin, Huijuan Cao, Shaoquan Zhang, Junfeng Chen, Jing Zhang, Juan Gao, Ildiko Gyory, Zhiliang Gao, Shiqiu Xiong, Bingliang Lin

**Affiliations:** ^1^ Department of Infectious Diseases, The Third Affiliated Hospital of Sun Yet-Sen University, Guangzhou, China; ^2^ Guangdong Provincial Key Laboratory of Liver Diseases, The Third Affiliated Hospital of Sun Yat-Sen University, Guangzhou, China, zssy.com.cn; ^3^ Department of Infectious Disease, The Third People’s Hospital of Shenzhen, Shenzhen, China, szdsyy.com; ^4^ Department of Molecular and Cell Biology, University of Leicester, Leicester, UK, le.ac.uk

**Keywords:** acute-on-chronic liver failure, bone marrow mesenchymal stem cell, GM-CSF, natural killer cell

## Abstract

**Background and Aims:**

Hepatitis B virus (HBV)‐related acute‐on‐chronic liver failure (HBV‐ACLF) is a life‐threatening syndrome characterized by rapid hepatic decompensation and immune dysregulation, with limited treatment options. Mesenchymal stem cells (MSCs) have shown therapeutic potential, but their precise mechanisms—particularly regarding their interaction with hepatic NKp46^+^DX5^−^ natural killer (NK) cells—remain unexplored. We investigated whether MSC treatment alleviates ACLF, which is associated with the modulation of NK cell populations, and sought to identify key factors driving this immunoregulatory effect.

**Methods:**

Peripheral NK cells were analyzed in ACLF patients receiving MSC infusion or standard therapy. Flow cytometry assessed NK frequency, phenotype, and correlations with survival. Murine ACLF was induced by carbon tetrachloride (CCL_4_), followed by MSC treatment. Flow cytometry characterized NK subsets (DX5^+/−^). In vitro cocultures evaluated MSC‐NK interactions, proliferation, and cytokine secretion. NK‐MSC conjugation was quantified via live imaging.

**Results:**

Frequency of peripheral blood NK cells from ACLF patients was upregulated after MSCs infusion, which correlated with liver function recovery. Phenotypic changes included upregulation of NKG2A and downregulation of NKp30 and FasL. MSC infusion effectively rescued ACLF mice and increased NK cell frequency in both blood and liver. Strikingly, NKp46^+^ DX5^−^, instead of NKp46^+^ DX5^+^ hepatic NK cells, responded well to MSCs. In vitro, MSCs promoted proliferation, granulocyte‐macrophage colony‐stimulating factor (GM‐CSF) secretion, and adhesion of NKp46^+^ DX5^−^ liver NK cells.

**Conclusions:**

MSC infusion holds significant clinical promise for ACLF patients. In addition to promoting liver regeneration, the immunomodulatory effect of MSCs on hepatic NKp46^+^DX5^−^ NK cells may provide a novel rationale for refining future therapeutic regimens.

## 1. Introduction

Hepatitis B virus (HBV)‐related acute‐on‐chronic liver failure (HBV‐ACLF) is a critical clinical syndrome marked by rapid hepatic decompensation in patients with pre‐existing chronic liver disease [1, 2]. Characterized by massive hepatocyte necrosis, excessive systemic inflammation, and high mortality rates (45% at 28 days and over 50% at 90 days globally), ACLF poses a major therapeutic challenge [3, 4]. The pathogenesis involves a complex interplay of immune dysregulation, hepatocyte necrosis, and inflammatory cascade activation, with innate immune hyperactivation playing a central role [5]. Notably, the COSSH study highlighted that excessive immune responses triggered by HBV drive disease progression from chronic hepatitis or cirrhosis to ACLF, positioning immune‐metabolic disturbances as a key axis in its pathophysiology [6].

Mesenchymal stem cells (MSCs) have emerged as a promising therapeutic candidate for ACLF due to their dual capacity for immunomodulation and tissue regeneration [7–9]. Clinically validated in diverse inflammatory conditions, including graft‐versus‐host disease (GVHD), myocardial infarction, and acute lung injury, MSCs have demonstrated efficacy in mitigating inflammation and promoting repair [10–12]. In our randomized controlled trial, MSCs therapy improved 24‐week survival (73.2% vs. 55.6%, *p* = 0.03) and led to greater reductions in Model for End‐Stage Liver Disease (MELD) scores at week 4 (3.1 ± 4.3 versus 0.4 ± 5.5, *p* = 0.004) in ACLF patients [13]. However, the underlying mechanisms remain poorly defined, necessitating deeper investigation into MSC–innate immune cell interactions to refine treatment strategies and elucidate ACLF pathogenesis.

A growing body of evidence demonstrates the immunomodulatory capacity of MSCs, particularly their regulatory effects on innate immune cells such as macrophages and natural killer (NK) cells [14, 15]. In the liver, NK cells constitute 30%–50% of the lymphocytes and play a pivotal role in hepatic immunity. Liver‐resident NK cells exhibit distinct phenotypes and functions compared to their counterparts in peripheral blood and the spleen [16, 17]. While in vitro studies demonstrated that MSCs suppress peripheral NK cell activation by dampening stimulatory receptors and interferon‐gamma (IFN‐γ) production [18, 19], their effects on tissue‐specific NK subsets within the inflamed liver microenvironment remain essentially unexplored. Building on our previous discovery that MSCs confer survival benefits in murine ACLF by driving M2 macrophage polarization via the Mertk‐JAK1/STAT6 axis [20], we hypothesize that MSCs may additionally exert therapeutic effects through selective modulation of liver‐resident NK populations. We thus hypothesized that MSCs alleviate ACLF by selectively modulating liver‐resident NK cell populations. This study aims to characterize MSC‐induced NK cell alterations in HBV‐ACLF patients and define the mechanistic basis of MSC‐NK crosstalk using a murine ACLF model.

Our work strives not only to expand the mechanistic understanding of MSCs therapy in ACLF but also to identify novel therapeutic targets for this devastating condition.

## 2. Materials and Methods

### 2.1. Clinical Study and Peripheral Blood NK Cell Characterization

The clinical trial protocol evaluating allogeneic bone marrow‐derived MSCs (allogeneic BM‐MSCs) treatment in HBV‐ACLF patients was previously reported [13]. All patients received standard medical treatment (SMT), including supportive care, antiviral therapy (entecavir 0.5 mg/day), and management of complications, such as infection, encephalopathy, and hepatorenal syndrome. In addition to SMT, patients in the MSC group received allogeneic BM‐MSCs intravenously at a standardized dose of 5.0 × 10^5^ cells/kg, which was selected following a preliminary dose‐finding phase once weekly for 4 weeks.

Allogeneic MSCs were isolated from bone marrow donated by healthy volunteers, aged 18–25 years old. They were free of hepatitis A, B, C, D, E viruses, human immunodeficiency virus (HIV), and syphilis infection and absence of hematologic diseases, such as thrombocytopenia and coagulation disorders. Bone marrow was obtained from iliac crests of healthy volunteers and diluted by 1:1 with phosphate buffered saline (PBS). Following current good manufacturing practices, mononuclear bone marrow cells were isolated by Ficoll–Hypaque (1.077 g/mL, HuaJing Bio‐Tech Co., Ltd., Shanghai, China) centrifugation. Cells were cultured at a density of 1 million mononuclear cells per milliliter in low glucose Dulbecco’s modified Eagle medium (L‐DMEM) (Hyclone, Logan, Utah) supplemented with 10% fetal bovine serum (FBS). The medium was changed every 3 days. Upon reaching 70%–80% confluence, MSCs were passaged using trypsin treatment. Cells were harvested at passage 5–6 and frozen in liquid nitrogen. Prior to injection, the cells were thawed and washed. Phenotypic characterization was performed by flow cytometry (markers CD29^+^/CD44^+^/CD73^+^/CD90^+^/CD105^+^/CD166^+^/CD45^−^/CD34^−^), while differentiation capacity was assessed using standard adipogenic and osteogenic induction protocols. Following sterility testing and quality control assessment, the cells were administered to patients via an intravenous injection. Participants were sequentially enrolled based on the admission time and allocated to either SMT group (*n* = 14) or MSC treatment (MSC group, *n* = 21). The study protocol complied with the Declarations of Helsinki and Istanbul Declaration and was approved by the ethics committee of the third affiliated hospital of Sun Yat‐Sen University (Approval Number YW2023‐004‐02), all participants provided written informed consent prior to enrollment in the study. Peripheral blood mononuclear cells (PBMCs) were isolated from the patient at baseline (week 0) and at weeks 2, 4, 8, 12, and 24 posttreatment. Thirteen healthy volunteers served as controls. For each sample, 1 × 10^6^ PBMCs were stained with antibody cocktails for surface marker analysis (Table S1A).

Flow cytometry was performed using an LSRII flow cytometer (BD Biosciences). Instrument setup and performance tracking utilized CS&T calibration beads (BD #661414), while rainbow calibration particles (BD #559123) were used for PMT voltage optimization. Fluorescence minus one (FMO) controls were included for accurate gating, and fluorochrome compensation was performed using anti‐rat IgG‐negative control (NC) compensation beads (BD #552845). Data analysis was conducted using FlowJo software (v10.0).

### 2.2. Carbon Tetrachloride (CCL_4_) Induced ACLF Murine Model

All experiments used 4–6‐week‐old male BALB/c mice (SPF grade) and were approved by the Animal Ethics Committee of Experimental Animal Ethics Committee of South China Agricultural University (Approval Number 2024h052). Mice were maintained under standard conditions (12 h light/dark cycle; allowed unlimited access to food/water) and randomly divided into four groups: (1) control, (2) ACLF (CCL_4_‐induced model), (3) ACLF + saline (saline‐treated), and (4) ACLF + MSC (MSC‐treated). CCL_4_ (Sigma, 319961) was diluted in olive oil (Sigma, 01514) to a final concentration of 10% (v/v) and 50% (v/v), respectively. Mice were administered 10% CCL_4_ (5 mL/kg, intraperitoneally) twice weekly for 8 weeks to induce liver cirrhosis, with acute decompensation triggered by a single sub‐lethal dose of 50% CCL_4_ (8 mL/kg, intraperitoneally) on day 3 of week 8. Control mice received equivalent volumes of olive oil [21]. Mice were deeply anesthetized with sodium pentobarbital (80 mg kg^−1^, i.p.). Under deep anesthesia, terminal blood collection was performed via the retro‐orbital sinus. Immediately following blood collection, the mice were euthanized by cervical dislocation to ensure death. All animal carcasses were collected and transferred to a certified medical waste disposal facility for proper incineration, strictly preventing any environmental exposure. Blood was collected at 0, 6, 12, 24, and 48 h after sub‐lethal CCL_4_ administration, centrifuged (2000 rpm, 5 min), and serum analyzed for ALT/AST using a Cobas 8000 analyzer (Roche). Liver tissues were fixed in 4% paraformaldehyde, paraffin‐embedded, and sectioned (5 μm). Sections were stained with hematoxylin (Sigma#MHS16) and eosin (Sigma#318906) for histopathology. The collagen fiber was stained with Sirius red (Sigma#365548).

### 2.3. Isolation and Identification of Bone Marrow MSCs

Bone marrow MSCs were isolated from 4–6‐week‐old BALB/c mice, as previously described [20]. Upon reaching 80% confluence, ~0.5 × 10^6^ cells per passage were trypsinized, washed, and resuspended in FACS buffer for phenotype analysis using antibody panels (Table S1B). Cell viability was assessed by live/dead staining.

### 2.4. Mononuclear Cell Isolation and NK Cell Characterization

PBMCs were isolated by density centrifugation using OptiPrep. Splenocytes were obtained through mechanical dissociation, followed by Ficoll enrichment. Liver mononuclear cells were isolated after perfusion with ice‐cold PBS, tissue digestion (0.05% collagenase IV [Sigma #C5138] + 0.001% DNase I [Sigma #11284932001], 37°C, 30 min), and Percoll gradient centrifugation. NK cell populations were characterized using optimized antibody panels (Table S1C).

### 2.5. MSCs Transplantation

ACLF mice received either 0.5 × 10^6^ MSCs suspended in saline or saline alone (control) via tail vein injection 1 h post‐CCL_4_ administration (*n* = 10/group).

### 2.6. In Vivo Tracking of MSCs Homing

To evaluate MSCs homing to target organs, BM‐MSCs were isolated from GFP‐transgenic C57BL/6 mice and administered to ACLF BALB/c mice via tail vein injection, as described above. At 6 and 24 h posttransplantation, mice were euthanatized, and the liver, spleen, and kidneys were rapidly harvested. Ex vivo fluorescence imaging was immediately performed using a high‐sensitivity Andor iKon CCD camera in the epi‐fluorescence mode (excitation: 465 nm; emission: 520 nm; exposure time: 8 s).

### 2.7. Hepatocellular Regeneration Assessment

Bromodeoxyuridine (BrdU, 75 mg/kg; Sigma) was administered intraperitoneally after MSCs transplantation, with a second dose given at 0, 12, or 24 h before sacrifice. Liver sections underwent antigen retrieval (microwave), blocking (5% goat serum), and staining with anti‐BrdU (Abcam #ab6326), followed by DAPI counterstaining.

### 2.8. Isolation of Hepatic NK Cell Subsets and In Vitro Stimulation

BALB/c mice were randomly assigned to receive either MSCs or an equal volume of saline (vehicle control). Forty‐eight hours posttreatment, livers were harvested under sterile conditions. Hepatic mononuclear cells were isolated via perfusion with ice‐cold PBS and enzymatic digestion, followed by Percoll density gradient centrifugation.

Liver lymphocytes were stained with a cocktail of fluorochrome‐conjugated antibodies against CD3 (BioLegend #100203), CD19 (BioLegend #115505), CD14 (BioLegend #123307), NKp46 (BioLegend #137608), and CD49b (DX5; BioLegend #108907). A fixable viability dye (Zombie; BioLegend #423101) was included to exclude dead cells. NK cell subsets—defined as live (Zombie^−^), lineage‐negative (CD3^−^CD19^−^CD14^−^), NKp46^+^DX5^−^, and NKp46^+^DX5^+^—were sorted at high purity (>95%) using a FACSAria II flow cytometer (BD Biosciences).

For stimulation assays, 96‐well tissue culture plates were precoated with the anti‐NKp46 antibody (clone 29A1.4; Invitrogen #16‐3351‐81) for 4 h at 37°C. Plates were washed three times with PBS before use. Sorted NK cells from both MSC‐ and saline‐treated groups were resuspended in RPMI‐1640 medium supplemented with 10% FBS and recombinant mouse interleukin (IL)‐15 (10 ng/mL; PeproTech). Cells were seeded (*n* = 6 biological replicates per group) under one of the following conditions: (i) IL‐15 alone (NC); (ii) IL‐15 plus plate‐bound anti‐NKp46 agonistic antibody; or (iii) IL‐15 combined with PMA (50 ng/mL) and ionomycin (1 μg/mL) as a positive control.

After 24 h of incubation at 37°C in 5% CO_2_, cell culture supernatants were collected to quantify granulocyte‐macrophage colony‐stimulating factor (GM‐CSF), IL‐2, IFN‐γ, and tumor necrosis factor‐alpha (TNF‐α) using commercial ELISA kits per the manufacturers’ instructions. Concurrently, total RNA was extracted from the cells using the Quick‐RNA Microprep Kit (ZYMO #R1050). RNA (10 ng) was reverse‐transcribed into cDNA, and gene expression levels of *Csf2* (GM‐CSF), *Il2*, *Ifng*, and *Tnf* were assessed by quantitative real‐time PCR (qRT‐PCR) the SYBR Green Master Mix. Relative mRNA expression was normalized to *Gapdh* and calculated using the 2^−ΔΔCt^ method.

### 2.9. MSCs Induced NK Cell Proliferation Assay

With NKp46‐Percp‐Cy5.5 (BD, 560800), DX5‐PE‐CF594 (BD, 562453), and live/dead cell fixable marker (Thermo Fisher, L34957), NK cell subpopulations (peripheral blood NKp46^+^ cells and liver NKp46^+^DX5^+/−^ cells) were purified by fluorescence‐activated cell sorting (FACSAria II, BD Biosciences) from normal and ACLF model mice. To obtain sufficient cell numbers, NK cells from 3 to 5 mice were pooled to obtain sufficient numbers. Subsequently, 0.5 × 10^6^ purified NK cells per well were plated in 24‐well U‐bottom plates and maintained in a complete medium supplemented with 100 U/mL IL‐2 (Pepro Tech) to support viability. After overnight incubation (37°C, 5% CO_2_), cells were labeled with a membrane‐permeant fluorescent proliferation dye (Abcam, #ab176736) according to the manufacturer’s protocol. For coculture, NK cells were combined with MSCs at a 1:1 ratio for 48 h, while control NK cells were cultured alone. Following incubation, cells were stained with NKp46‐Percp‐Cy5.5 (BD, 560800) and live/dead cell fixable marker (Thermo Fisher, L34957), and run samples on LSRII flow cytometer (BD Biosciences).

### 2.10. MSC‐Mediated NK Cell Activation Assessment

1 × 10^6^ liver NKp46^+^DX5^+/−^ cells were purified as above and then transferred into 24‐well flat‐bottom plates, with or without an 80% confluent MSCs feeder layer at a 1:1 ratio. After 12 h of coculture, 6 μg/mL GolgiStop (BD Biosciences, 554724) was added. In another 4 h before suspension, the cells were harvested and stained with NKp46‐Percp‐Cy5.5 (BD, 560800), DX5‐PE‐CF594 (BD, 562453), CD3‐APCcy7 (Biolegend, 100221), CD107a‐brilliant violet 421 (Biolegend, 328626), and live/dead cell fixable marker (Thermo Fisher, L34957). Then, cells were fixed, permeabilized, and stained with IFN‐r‐APC (Biolegend, 505810) and GM‐CSF‐FITC (Biolegend, 505404). Samples were analyzed on an LSRII flow cytometer to assess activation markers and cytokine production.

### 2.11. NK Cell‐MSC Conjugation Assays

Liver NK cells and MSCs were differentially labeled with PKH26 (Sigma–Aldrich #PKH26PCL) and PKH67 (Sigma–Aldrich #PKH67GL) membrane dyes, respectively, following the manufacturer’s instructions. Labeled cell suspensions (0.1 × 10^6^ cells each) were combined in 50 μL medium in 96‐well V‐bottom plates, briefly centrifuged (300 × *g*, 1 min) to initiate contact, and incubated for 30 min at 37°C. Reactions were terminated by adding ice‐cold 1% paraformaldehyde. Conjugate formation was quantified by dual‐positive fluorescence using flow cytometry.

### 2.12. Statistical Analysis

Statistical analyses were performed using SPSS 22.0 software (IBM Corp., Armonk, NY, USA). Data are presented as the mean ± SD (continuous) or frequencies (categorical). Normality was assessed using the Shapiro–Wilk test. For paired comparisons, paired *t*‐tests or Wilcoxon tests were used, as appropriate. Survival data were analyzed by Kaplan–Meier with the log‐rank test. Correlations were used by Spearman’s test. A two‐tailed *p*  < 0.05 was considered statistically significant.

## 3. Results

### 3.1. MSCs Therapy Expands Peripheral NK Cells Associated With Survival in HBV‐ACLF Patients

**Figure 1:**
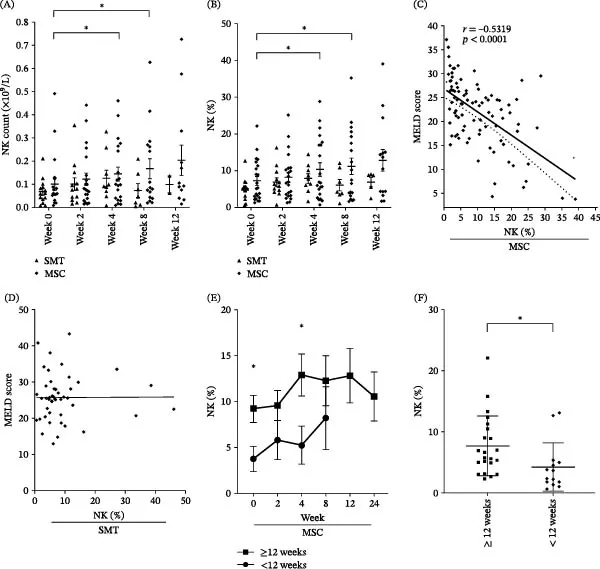
NK cell expansion following MSC therapy reflects survival benefits in HBV‐ACLF. (A, B) Absolute counts of peripheral blood NK cells (CD3^−^CD56^+^) in MSC‐treated (•) and SMT (▲) groups, measured at baseline and follow‐up timepoints. Both absolute NK cell counts and NK cell proportions (NK%) were significantly increased at weeks 4 and 8 compared to baseline in MSC‐treated group, but remained unchanged in the SMT group. Data are presented as mean ± SD.  ^∗^ *p*  < 0.05 (paired *t*‐test). (C) Correlation between NK cell proportions and MELD scores in the MSC‐treated group (Pearson’s *r* = −0.5319, *p*  < 0.0001, *n* = 21 patients). The solid line represents the linear regression fit, with dashed lines indicating the 95% confidence interval (CI). (D) No significant correlation between MELD score and NK cell proportion in SMT group (Pearson’s *r* = 0.005527, *p*  = 0.9709, *n* = 14 patients). (E) Long‐term survivors (≥12 weeks, *n* = 13 patients) exhibited significantly higher peripheral NK cell proportions at baseline and week 4 compared to short‐term survivors (<12 weeks, *n* = 8 patients) within the MSC‐treated cohort. Data are presented as mean ± SD.  ^∗^ *p* < 0.05 (unpaired *t*‐test). (F) Combined data from both MSC‐treated and SMT groups show higher baseline NK cell frequencies in patients surviving ≥12 weeks (*n* = 22 patients) compared to those surviving <12 weeks (*n* = 14 patients). Data are analyzed using Student’s *t*‐test, presented as mean ± SD.  ^∗^ *p* < 0.05 (unpaired *t*‐test). NK cell counts were quantified as absolute numbers within the total lymphocyte population. NK (%) indicates the frequency of CD56^+^ CD3^−^ NK cells among peripheral blood lymphocytes. Each point corresponds to a single patient at a posttreatment timepoint. Data are representative of at least three independent experiments.

In our pilot clinical trial evaluating allogeneic BM‐MSCs in patients with HBV‐ACLF, peripheral blood immune cell counts were monitored throughout the treatment period. Systematic immune cell profiling revealed that MSC therapy selectively expanded peripheral NK cells, increasing both their absolute counts and proportions, whereas the standard medical therapy (SMT) showed no significant effect on NK cell dynamics (Figure 1A, B). Meanwhile, no significant changes were observed in non‐NK cell populations (Figure S1B, C), peripheral neutrophil counts declined similarly in both groups (Figure S1D, E).

Notably, MSC‐induced NK cell expansion demonstrated significant clinical relevance. In the MSC‐treated group, NK cell proportions were negatively correlated with MELD scores (Figure 1C), whereas the SMT showed no correlations (Figure 1D). In the MSC‐treated cohort, higher baseline NK cell proportions correlated with prolonged survival (≥12 weeks; Figure 1E). This association was also observed in pooled baseline data from both the MSC‐treated and SMT groups: patients surviving ≥12 weeks consistently exhibited higher NK cell frequencies than those who died before 12 weeks (<12 weeks; Figure 1F). These findings suggest that NK cells may contribute to MSC‐mediated therapeutic benefits, and their frequency could represent a potential prognostic biomarker.

### 3.2. MSCs Treatment Modulates NK Cell Phenotype and Functional Regulation

**Figure 2:**
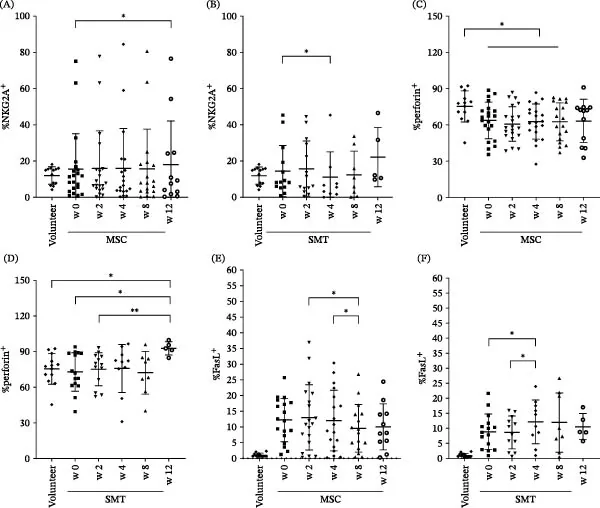
MSC treatment upregulates NKG2A while suppressing cytotoxic markers. (A, B) MSC‐treated patients showed progressive upregulation of NKG2A level by week 12 compared to baseline, whereas the SMT group exhibited an early decline at week 4 versus baseline. (C, D) MSC‐treated patients showed persistently lower perforin level across all timepoints compared to healthy controls. In contrast, SMT patients demonstrated a late increase in perforin by week 12 versus baseline. (E, F) FasL levels were elevated in both groups compared with healthy controls. MSC‐treated patients exhibited progressive FasL reduction, reaching significance by week 8 (vs. weeks 2/4), whereas SMT group displayed increased FasL at week 4 (vs. weeks 0/2). %NKG2A^+^/%perforin^+^/%FasL^+^ indicates frequency of NKG2A/perforin/FasL positive cells among peripheral blood mononuclear cells (PBMCs). Each point represents data from one patient. Data are analyzed using Student’s *t*‐test, presented as mean ± SD (*n* = 21/MSC group, *n* = 14/SMT group).  ^∗^ Indicate *p* < 0.05,  ^∗∗^ indicate *p* < 0.01. Data are representative of at least three independent experiments.

To unveil the potential mechanism by which MSC infusion induced specific changes in NK cells, their phenotypic and functional markers were comprehensively characterized. Among all of them, the percentage of NKG2A^+^ cells increased by week 12 in the MSC‐treated group (Figure 2A), whereas the SMT‐treated group exhibited a decline in NKG2A expression at week 4 (Figure 2B). On the contrary, MSC treatment led to a progressive downregulation of cytotoxic marker perforin and FasL expression in peripheral blood NK cells over a 12‐week period (Figure 2C, E). Conversely, the SMT group exhibited an increased expression of both markers during treatment (Figure 2D, F). No significant changes were observed in activating receptors NKG2D, NKp46, and NKp30 in both groups. (Figure S2). Inhibitory KIR receptors KIR2DL1 and KIR2DL3 also remained unchanged, and KIR3DL1 expression demonstrated gradual recovery in both groups (Figure S3). At baseline, no statistically significant differences were observed in these markers between the MSC‐treated and standard therapy groups.

### 3.3. Transplanted MSCs Rescue ACLF Mice and Promote Liver Regeneration

**Figure 3:**
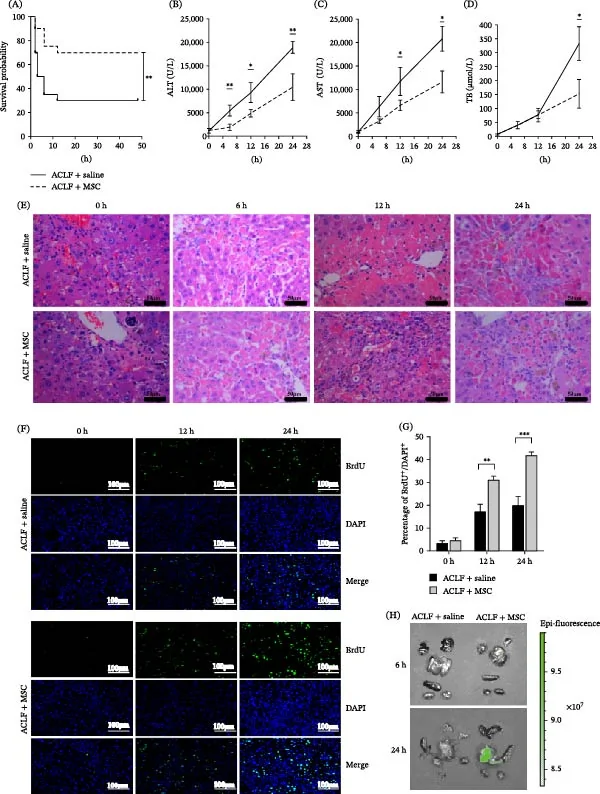
Transplanted MSCs ameliorate liver injury and promote liver regeneration in ACLF mice. (A) Kaplan–Meier analysis revealed a significantly prolonged survival in MSC‐treated mice compared to saline‐treated mice (*n* = 10 mice /group). Log‐rank *χ* ^2^ = 7.36, *p* = 0.0067. (B–D) The serum liver function markers (ALT/AST/TB) from mice with/without MSCs administration at 6, 12, 24 h posttreatment. (E) H&E staining of liver sections from ACLF mice at 0, 6, 12, and 24 h posttreated with saline or MSCs. In the saline group, progressive necrosis and hemorrhage (red blood cell extravasation) worsened over 24 h. In the MSC group, necrosis and hemorrhage were significantly attenuated by 12 h, with further improvement at 24 h. Similar results were obtained from three individual experiments. (F) Immunofluorescence staining of BrdU labeled liver from ACLF mice with/without MSC transplantation. Nuclei were counterstained with DAPI (blue). BrdU‐positive cells (green, active DNA synthesis) were rare in saline controls but detectable in MSC‐treated livers by 12 h and further increased at 24 h. Images are representative of three replicates. (G) MSC treatment significantly increased BrdU^+^/DAPI^+^ cell ratios. Quantitative analysis of BrdU^+^ cells per high‐power field (HPF). Percentage of BrdU^+^/DAPI^+^ refers to the proportion of BrdU‐positive cells among all DAPI‐positive cells. (H) Distribution of GFP‐labeled MSCs in organs, strong GFP fluorescence signals were detected in the liver of the MSC‐treated group (ROI = 3.298 × 10^9^ p/sec/cm^2^/sr). In contrast, weaker and diffuse signals were observed in the liver from saline treatment group (ROI = 1.629 × 10^9^ p/sec/cm^2^/sr), and minimal signals were detected in the spleen and other tissue. Data are analyzed using Student’s *t*‐test and are presented as mean ± SD (*n* = 10 mice/group),  ^∗^ *p* < 0.05,  ^∗∗^ *p* < 0.01,  ^∗∗∗^ *p* < 0.001. Data are representative of at least three independent experiments.

To elucidate the underlying mechanism, we established a murine model of ACLF (Figure S4A). The model showed high lethality, with 70% mortality (7/10 mice) within 48 h after the drug challenge versus no deaths in control animals (Figure S4B). In ACLF mice, serum ALT and AST levels were significantly elevated at 24 h compared to the NC group (*p*  < 0.001 for both; Figure S4C). Histological analysis confirmed background cirrhosis via sirius red staining. Furthermore, the liver exhibited massive hepatic necrosis characterized by near‐complete loss of hepatocytes throughout the lobules, accompanied by residual intrasinusoidal inflammation, periportal ductular reaction (neocholangiolar proliferation), and inflammatory infiltration in the portal and periportal areas (Figure S4D). Murine BM‐MSCs were isolated from bone marrow, expanded in culture, and verified by flow cytometry (Figure S5A,B). MSCs transplantation significantly improved survival within 48 h (*p* = 0.0067, Figure 3A). Compared to saline‐treated mice, MSCs administration resulted in a rapid decline in ALT levels at 6 h, followed by significant reductions in AST at 12 h and total bilirubin (TB) at 24 h (Figure 3B–D). This protective effect was evident early on, with hepatic necrosis beginning to resolve at 6 h and showing progressive improvement between 12 and 24 h (Figure 3E). Furthermore, strong GFP fluorescence was detected specifically in the liver—relative to the spleen and kidneys—indicating the effective homing of MSCs to the injured hepatic tissue (Figure 3H). Notably, BrdU‐labeled hepatocytes markedly increased at 12 and 24 h post‐MSCs infusion, indicating enhanced liver regeneration (Figure 3F, G).

### 3.4. MSCs Expand the DX5^−^ Liver NK Cell Population and Modulate Their Phenotype In Vivo

**Figure 4:**
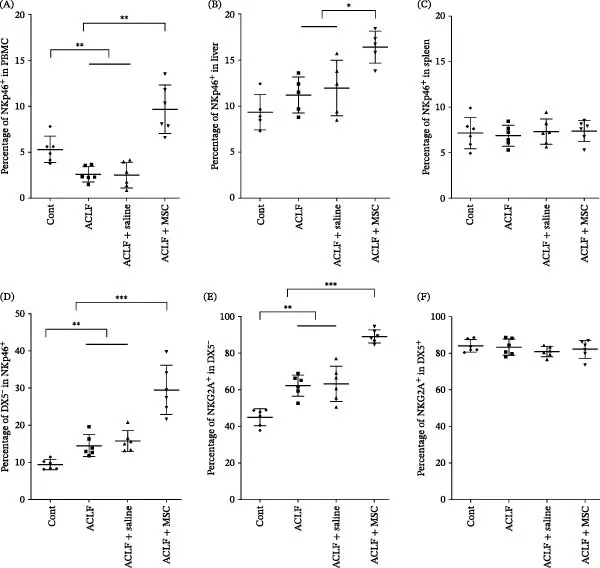
MSCs selectively expand liver DX5^−^ NK cells and modulate their phenotype in ACLF mice. (A–C) Frequencies of NKp46^+^ NK cells in PBMCs, liver, and spleen from untreated controls (Cont), untreated ACLF mice (ACLF), and ACLF mice treated with saline or MSCs for 48 h. (A) Significantly reduced NKp46^+^ NK cells in PBMCs of ACLF mice compared to normal controls, which was significantly reversed by MSC transplantation. (B) No significant difference in liver NKp46^+^ NK cells was observed between ACLF and control groups, but MSC treatment further increased their frequency. (C) Splenic NKp46^+^ NK cells remained stable across all groups. (D) ACLF mice exhibited a higher proportion of DX5^−^ NK cells (within NKp46^+^) than controls, MSC transplantation further amplified DX5^−^ NK cell expansion. (E) MSCs upregulated NKG2A on liver DX5^−^ to levels matching DX5^+^ NK cells (*p* < 0.01 vs. untreated ACLF). (F) No significant changes in NKG2A expression were observed on DX5^+^ cells across all groups. Percentage of NKp46^+^ in PBMC/liver/spleen indicates NKp46 positive cells among mononuclear cells in each compartment. Percentage of DX5^−^ in NKp46^+^ denotes the proportion of DX5^−^ cells among NKp46^+^ liver mononuclear cells. Percentage of NKG2A^+^ in DX5^−^/DX5^+^ indicates the frequency of NKG2A positive cells within each NK subset. Data are analyzed using Student’s *t*‐test and presented as mean ± SD (*n* = 6 mice/group).  ^∗^ *p* < 0.05,  ^∗∗^ *p* < 0.01,  ^∗∗∗^ *p* < 0.001. Data are representative of at least three independent experiments.

To validate these clinical findings, we investigated MSC‐mediated mechanisms in a murine ACLF model. MSC transfusion significantly increased NK cell frequency in blood and liver but not spleen (Figure 4A–C). Strikingly, MSC transfusion preferentially expanded the mice liver DX5^−^ NK subset (Figure 4D) and correspondingly decreased the frequency of DX5^+^ NK. The DX5^−^ liver NK typically exhibits a lower NKG2A expression than DX5^+^ NK cells in healthy controls (Figure S6A). As expected, MSCs treatment upregulated NKG2A expression in DX5^−^ NK cells to levels comparable to those of DX5^+^ NK cells (Figure 4E, F), which echoed our findings in human peripheral blood NK cells (Figure 2A). Additionally, MSC treatment increased the frequencies of Ly49A and Ly49C while downregulating NKG2D in the liver NK cells (Figure S6E–G). Notably, there were no significant differences in these markers between the DX5^−^ and DX5^+^ subsets under physiological conditions (Figure S6B–D).

### 3.5. MSCs Selectively Promote Proliferation and GM‐CSF Secretion in DX5^−^ Liver NK Cells In Vitro

**Figure 5:**
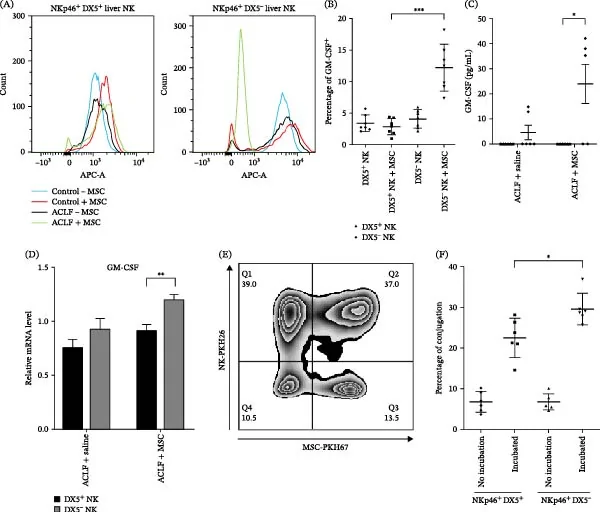
MSCs selectively promote proliferation and GM‐CSF secretion in DX5^−^ liver NK cells. (A) Sorted liver NKp46^+^DX5^+/−^ NK cells from normal mice coculture with/without MSCs (in blue and red), and from ACLF mice with/without MSCs (in green and black) were labeled with a proliferation dye and assessed after 48 h. MSC coculture significantly enhanced proliferation in DX5^−^ NK cells versus DX5^+^ cells from ACLF mice, overlaid histogram showed from one representative from three experiments. (B) Intracellular GM‐CSF staining in NKp46^+^DX5^+/−^ NK cells from ACLF mice with or without MSC treatment. MSC‐induced GM‐CSF secretion specifically in DX5^−^ NK cells. (C) ELISA measurement of GM‐CSF in supernatants of sorted hepatic NKp46^+^DX5^−^ and NKp46^+^DX5^+^ NK cells from MSC or saline‐treated ACLF mice after in vitro NKp46 crosslinking. Only DX5^−^ NK cells from MSC‐treated mice showed significant GM‐CSF secretion. (D) qRT‐PCR validation of *Csf2* mRNA upregulation in the same DX5^−^ subset. In saline‐treated controls, both subsets produced negligible GM‐CSF. Other cytokines (IFN‐γ, TNF‐α, and IL‐2) were undetectable under this stimulation condition. (E) Representative FACS plot of PKH26‐stained NK cells cocultured with PKH67‐stained MSCs: double‐positive events indicate cell conjugation. (F) Quantification of conjugation frequency (defined as PKH26+PKH67+ double‐positive events). DX5^−^ NK cells exhibited significantly higher conjugation frequency with MSCs than DX5^+^ NK cells (*p* < 0.05). Data are analyzed using unpaired *t*‐test, presented as mean ± SD (*n* = 12 mice /group).  ^∗^ *p* < 0.05,  ^∗∗^ *p* < 0.01,  ^∗∗∗^ *p* < 0.001. Data are representative of at least three independent experiments.

To elucidate the molecular machinery by which MSCs affect DX5^−^ and DX5^+^ liver NK cells, MSCs from inbred mice were cocultured in vitro with purified and fluorescence‐labeled DX5^−^, NKp46^+^DX5^+^ liver mononuclear cells isolated from ACLF mice and normal mice. Peripheral blood NK cells were also included as controls. Strikingly, MSCs significantly enhanced the proliferation of DX5^−^ liver NK cells from ACLF mice but not from normal mice. This proliferative effect was not observed in DX5^+^ liver NK cells from either ACLF or normal mice (Figure 5A). Furthermore, MSC coculture specifically augmented GM‐CSF expression in DX5^−^ liver NK cells but not in DX5^+^ counterparts (Figure 5B). Consistent with these functional differences, DX5^−^ liver NK cells exhibited stronger conjugation with MSCs compared to DX5^+^ liver NK cells. (Figure 5E, F).

To investigate the functional modulation of hepatic NK cell subsets by MSCs therapy in ACLF, we isolated NKp46^+^DX5^−^ and NKp46^+^DX5^+^ NK cells from the livers of MSC‐ or saline‐treated BALB/c mice 48 h posttreatment and subjected them to in vitro stimulation. Upon NKp46 crosslinking, DX5^−^ NK cells from the MSC‐treated group exhibited a marked increase in GM‐CSF secretion compared to DX5^+^ NK cells, as measured in culture supernatants by ELISA (Figure 5C). This finding was corroborated at the transcriptional level, where qRT‐PCR revealed a corresponding upregulation of *Csf2* mRNA in the same subset (Figure 5D).

In saline‐treated control mice, both DX5^−^ and DX5^+^ hepatic NK cell subsets exhibited negligible GM‐CSF production, with no significant difference observed between them. In stark contrast, IL‐2, IFN‐γ, and TNF‐α were undetectable or below the limit of quantification in all supernatants following NKp46 stimulation, irrespective of the treatment group or NK subset. Consistently, qRT‐PCR analysis revealed very low or absent mRNA expression of *Il2*, *Ifng*, and *Tnf* across all conditions.

As a positive control, stimulation with PMA and ionomycin induced potent cytokine responses, with DX5^−^ NK cells producing significantly higher levels of GM‐CSF, IFN‐γ, and IL‐2 compared to DX5^+^ NK cells in both treatment groups (Figure S7A–H), thereby confirming the intrinsically greater effector capacity of the DX5^−^ subset. No detectable levels of these cytokines were observed in the NC groups (IL‐15 alone) across all subsets and conditions.

## 4. Discussion

ACLF is characterized by acute deterioration of liver function in patients with pre‐existing chronic liver disease and is associated with high mortality. The pathogenesis of ACLF involves a cytokine storm that triggers systematic inflammatory responses and subsequent immune paralysis [22, 23]. BM‐MSCs transplantation has shown partial success in the clinical treatment of ACLF [24, 25], highlighting its potential as a therapeutic strategy. However, its therapeutic mechanisms remain incompletely understood.

MSCs exhibit unique immune‐privileged properties, immunomodulatory capacity, and tissue regeneration potential, making them promising candidates for ACLF therapy [26–28]. Our clinical trials demonstrated that allogeneic BM‐MSCs infusion is safe and improves the 24‐week survival rates in (HBV‐ACLF) patients by enhancing liver function and reducing severe infections [13]. However, the molecular and cellular mechanisms underlying these effects are far from being fully understood, and further research is urgently needed to improve clinical treatment strategies.

Due to the practical challenges of obtaining liver samples from ACLF patients, we started by analyzing patients’ peripheral blood as a surrogate tissue. We found that both the frequency and absolute count of peripheral blood immune cells were significantly reduced in ACLF patients compared to healthy volunteers, likely reflecting leukocytes redistribution to the liver during several and acute liver inflammation. Remarkably, MSC transfusion was not only correlated with inflammation suppression and liver regeneration but also specifically restored peripheral blood NK cell frequency and absolute count to near‐normal levels by week 8. Moreover, this increase in NK cell proportion was positively correlated with a better prognosis, as indicated by the MELD score and survival rate. These findings suggest that peripheral blood NK cell counts could serve as a novel biomarker for clinical MSCs treating ACLF patients and prompted us to investigate the underlying mechanisms.

To date, there is no strong evidence to support MSCs infusion induced hematopoietic stem cell (HSC)‐mediated NK cell development in bone marrow. It is also unlikely that MSC suppressed liver inflammation, which subsequently redistributed NK specifically from the liver to peripheral blood.

To find some clue of MSCs infusion on NK cells, we characterized typical phenotypic and functional markers of NK cells from ACLF patients during MSCs treatment. Interestingly, MSCs infusion upregulated the inhibitory receptor NKG2A while downregulating perforin and FasL, the markers of cytolytic function, in peripheral blood NK cells, suggesting a shift toward a more immature, regulated, and less cytotoxic NK cell population. Since transplanted MSCs have been shown to migrate to and reside in the liver [29–31], they may induce proliferation of liver NK cells in situ and/or modulate NK cell immune responses by fine‐tuning the balance between activation and inhibition signals to mitigate excessive inflammation.

To validate our hypothesis, we established CCL_4_‐induced murine ACLF models, which are widely used to mimic human ACLF conditions and accommodate MSCs treatment. Indeed, MSCs infusion significantly improved survival rates, alleviated liver damage, and promoted hepatic regeneration in ACLF mice, echoing our clinical observations in HBV‐ACLF patients treated with MSCs infusion. Although MSCs promote cell proliferation in the liver, it is unclear whether these were hepatocytes or immune cells. This led us to specifically investigate NK cells in ACLF pathogenesis.

Like clinical observations, MSCs infusion indeed reversed the decreased frequency of peripheral blood NKp46^+^ NK cells in ACLF mice, restoring them to above normal levels, and thereby confirmed the reliability of this mouse model. Interestingly, MSCs infusion elevated NKp46^+^ liver NK significantly in ACLF mice but not spleen. Considering bone marrow HSC‐mediated NK cell development normally circulates and resides in both liver and spleen, this differential response to MSCs infusion suggests MSCs interacted with liver NK cells in situ.

NK cells are the predominant innate immune cells and major hepatic leukocyte population in the liver, yet their physiological functions remain poorly understood [32, 33]. Our study demonstrated that MSCs infusion selectively expands NKp46^+^ NK cells in the liver but not in the spleen of ACLF mice. This spatial specificity likely arises from a synergistic interplay between MSCs tropism, hepatic immune niches, and microenvironmental cues. First, MSCs exhibit preferential homing to injured tissues, particularly fibrotic and inflamed livers. Ex vivo imaging revealed robust MSC engraftment in the liver at 6–24 h postinfusion, with minimal retention in the spleen or kidneys (Figure 3H). Second, once localized to the liver, MSCs engage in both direct and paracrine crosstalk with the resident immune cells, including NK subsets. This aligns with prior findings demonstrating that MSC administration enhances hepatic NK cell accumulation in liver fibrosis, possibly due to local cytokine recruitment, while sparing splenic NK [32].

Third, the liver microenvironment is enriched with a complex cytokine milieu such as IL‐7, IL‐15, IL‐12, IL‐18, and IL‐2, all of which are known to promote the expansion and functional maturation of immature or tissue‐adapted NK cells [33]. Fourth, emerging evidence suggests that liver‐resident NK progenitor cells retain the capacity to proliferate and differentiate locally in response to inflammatory signals [34]. Importantly, MSCs therapy may preferentially amplify this intrahepatic regenerative pathway—potentially through provision of niche‐supportive factors (such as IL‐7, SCF, IL‐21, and PGE_2_)—thereby obviating the need for de novo recruitment of NK cells from peripheral circulation in ACLF [35]. In contrast, splenic NK cell responsiveness may be intrinsically blunted in ACLF due to systemic immunosuppression, sequestration, or exhaustion—a hallmark of advanced liver failure [36].

Emerging evidence classifies liver NK cells into conventional circulating NK cells and resident NK cells based on their origin pathways, gene and protein expression profiles, and functional characters. CD49a^+^DX5^+^ are conventional circulating NK cells derived from bone marrow HSCs. However, CD49a^+^DX5^–^ originate from intrahepatic HSCs or progenitor cells and reside in hepatic sinusoids, playing a critical role in homeostasis maintenance and rapid response to microenvironment perturbations [37, 38]. Notably, we demonstrated for the first time that MSC infusion upregulated liver NKp46^+^DX5^–^ cells alongside increased their NKG2A expression. Previous in vitro studies have demonstrated context‐dependent MSC‐NK cell interactions influenced by MSC source, MSC‐to‐NK cell ratios, and microenvironmental factors [39–41], guiding our investigation into MSCs’ effects on NK cells in ACLF.

In vitro coculture of purified NKp46^+^DX5^+^ and NKp46^+^DX5^−^ with MSCs confirmed that MSCs specifically promote NKp46^+^DX5^−^ cell proliferation (but not NKp46^+^DX5^+^), which may be due to the higher conjugation capacity between NKp46^+^DX5^-^ and MSCs than NKp46^+^DX5^+^ counterparts. This MSC‐induced NKp46^+^DX5^−^ proliferation is consistent with the increased frequency of liver NK cells.

Meanwhile, MSCs specifically enhanced GM‐CSF production by liver NKp46^+^DX5^−^ cells specifically. GM‐CSF, traditionally recognized as a hematopoietic growth factor, has been implicated in biliary wound healing and hepatocellular regeneration [42, 43] and modulation of lymphoid/myeloid populations in inflammatory/autoimmune conditions [44, 45]. Given the shared immunological features between sepsis and ACLF, our findings suggest that DX5^−^ liver NK‐derived GM‐CSF may contribute to liver regeneration in ACLF, potentially improving clinical outcomes.

Interestingly, growing evidence demonstrates that GM‐CSF reciprocally promotes MSCs migration and proliferation during inflammation [46, 47], suggesting a positive feedback loop: MSC‐primed NK cells secrete GM‐CSF, which in turn enhances MSCs recruitment and expansion. An upcoming in vivo NK depletion assay will be conducted to confirm the current in vitro data. Although our current data focus on NK cell‐intrinsic GM‐CSF responses, we acknowledge that MSC‐derived factors, which are known to modulate NK cell adhesion and survival, might synergistically enhance this effect. Future studies employing MSC‐conditioned media or cytokine blockade could clarify whether these mediators potentiate the observed GM‐CSF secretion.

While the CCl_4_‐induced model is useful for studying general mechanisms of liver injury, fibrosis, and repair, it lacks virological, immunological, and pathophysiological relevance to HBV‐ACLF. For translational research, combining this model with HBV‐replicating systems or moving toward humanized mouse models may provide more clinically relevant insights.

Regarding the identification of liver‐resident NK cells, we acknowledge a limitation in this study. Although the NKp46^+^DX5^−^ subset is often considered representative of liver‐resident NK cells, this phenotypic definition alone may not fully distinguish them from other hepatic innate lymphoid cells (ILCs) or transitional NK cell stages [48]. To ensure scientific rigor, we have conservatively referred to these cells as ‘hepatic NKp46^+^DX5^−^ NK cells’ throughout the Results section. Future studies utilizing additional resident markers (such as CXCR6 or CD49a) or single‐cell sequencing are warranted to precisely confirm the residency status and developmental origin of this subset in the context of ACLF.

## 5. Conclusion

Our findings innovatively establish MSCs as pivotal regulators of hepatic NKp46^+^DX5^−^ NK cell activity and promote their proliferation and GM‐CSF secretion, which resulted in immunomodulation, tissue repair, and longer survival in the complex milieu of ACLF. MSCs represent promising therapeutic candidates for liver failure management. These discoveries reveal novel MSC‐NK interactions and provide a scientific foundation for advancing ACLF treatment and novel biomarkers for prognosis of disease monitoring.

## Author Contributions

**Bingliang Lin:** conception, design, financial support, provision of study material or patients. **Jing Xiong:** conception, design, performed experiments, data analysis, interpretation, manuscript writing. **Shiqiu Xiong:** conception, design, data analysis, interpretation, manuscript writing. **Weizhen Weng and Dengna Lin**: performed experiments. **Juan Gao:** sample collection. **Zhiliang Gao:** conception, design, financial support. **Huijuan Cao and Shaoquan Zhang:** provision of study material and patients. **Junfeng Chen**: collection and assembly of data. **Jing Zhang:** provision of patients. **Ildiko Gyory:** revise the manuscript.

## Funding

This work was supported by the National Key Research and Development Program of China (Grant 2024YFA1108802), the National Natural Science Foundation of China (Grant 81901940), the Natural Science Foundation of Guangdong Province (Grant 2021A1515010306), and the Guangdong Basic and Applied Basic Research Foundation (Grant 2022A1515110769).

## Disclosure

An earlier version of this manuscript was published as a preprint on Research Square [49]. The current version includes additional data analysis and revised conclusions.

## Conflicts of Interest

The authors declare no conflicts of interest.

## Supporting Information

Additional supporting information can be found online in the Supporting Information section.

## Supporting information


**Supporting Information** Supporting Information File 1: Ethics approval documents and study protocols. This file contains the official ethical approval certificates (in both Chinese and English) and the corresponding study protocols for both the animal experiments and the human subject studies involved in this research. Supporting Information File 2: Health and weight monitoring. This document provides detailed data and protocols regarding the health and weight monitoring of the mice during the study. Supporting Information File 3: Author checklist. The completed checklist confirming that the manuscript adheres to the journal’s fundamental academic writing guidelines.

## Data Availability

The data that support the findings of this study are available from the corresponding author upon reasonable request.
