# Supplementary material for: Mesenchymal Stem Cells Ameliorate Acute‐on‐Chronic Liver Failure Associated With Expansion of Immunoregulatory Hepatic NKp46+DX5− NK Cells
Source: J Immunol Res. 2026 Sep 24;2026:2373331. doi: 10.1155/jimr/2373331 (PMC13602807; doi:10.1155/jimr/2373331)
Supplement: Supplementary file 1 [file JIMR-2026-2373331-s001.zip › Supplementary figure legends.docx]

**Supplement Figure legends**

**Supplementary Figure 1. Neutrophil Count and NK Cell Dynamics Changes.**

**(A)** gating strategy. Lymphocytes were selected by FSC/SSC. Doublets were excluded using FSC-H vs. FSC-A gating. NK cells were identified as CD3⁻CD56⁺ cells, with dead cells excluded via LIVE/DEAD™ Far-Red staining. The expression of NKG2A, Perforin, FasL, NKG2D, NKp30, NKp46, KIR2DL1, KIR3DL1, and KIR2DL3 was subsequently evaluated within this NK cell subset. **(B, C)** Frequency of non-NK lymphocytes remained unchanged in both MSC and SMT groups**. (D, E)** Neutrophil counts declined similarly over time in both groups. Non-NK count was defined as total lymphocytes count minus NK cells count. Data are analyzed using Student’s t-test, presented as mean ± SD (n =21 patients for MSC group, n=14 patients for SMT group). * P<0.05, ** P<0.01, *** P<0.001. Data are representative of at least three independent experiments.

**Supplementary Figure 2. Dynamic Changes in NK Cell Activating and Cytotoxic Markers during MSC therapy in HBV-ACLF patients.**

**(A, B)** NKG2D expression was downregulated in MSC-treated patients at week 8 versus week 2. In contrast, the SMT group showed No significant change in NKG2D. **(C, D)** NKp46 expression remained stable in the MSC group, whereas the SMT group exhibited transient suppression (Weeks 0–4) followed by recovery by Week 8. **(E, F)** NKp30 expression was significantly downregulated at week 12 compared to baseline in the MSC group. The SMT group exhibited no significant changes. %NKG2D^+^/ %NKp46^+^/ %NKp30^+^ indicates frequency of positive cells among PBMCs. Data are analyzed using Student’s t-test, presented as mean ± SD (n =21/MSC group, n=14/SMT group). * P<0.05, ** P<0.01. Data are representative of at least three independent experiments.

**Supplementary Figure 3. Kinetics of inhibitory KIR receptor expression in MSC and SMT groups.**

**(A–D)** KIR2DL1 and KIR2DL3 expression remained unchanged in both groups across all time points (*P* > 0.05). **(E, F)** KIR3DL1 expression was significantly lower in MSC group compared with healthy controls but gradual recovery after treatment, with earlier upregulation in the MSC group (Weeks 2 and 8) and delayed restoration in the SMT group (Weeks 8 and 12). % KIR2DL1^+^/ %KIR2DL3^+^/ %KIR3DL1^+^indicates frequency of positive cells among PBMCs. Data are analyzed using Student’s t-test, presented as mean ± SD (n =21/MSC group, n=14/SMT group). * P<0.05. Data are representative of at least three independent experiments.

**Supplementary Figure 4. Establishment of ACLF animal model**.

(A) Schematic of the experimental protocol. (B) Survival curve of mice challenged with sublethal CCl₄ (n=10 mice/group). In the ACLF model Group, 3 mice died within 2h, 2 at 3h and 2 at 6h. Representative of three independent experiments (log-rank χ^2^ =78.063, p=0.0045). (C) Serum transaminase levels: ALT and AST were increased in cirrhotic mice and markedly elevated in ACLF mice compared to oil-only controls. Data are analyzed using Student’s t-test, presented as mean ± SD (n=10 mice/group) (D) Upper row: H&E staining; Lower row: Sirius Red staining. Representative of three independent experiments. Data are analyzed using Student’s t-test, presented as mean ± SD (n=6/group). ** P<0.01, *** P<0.001. Data are representative of at least three independent experiments.

**Supplementary Figure 5. characterization of murine BM-MSCs and NK cell gating strategy.**

**(A)** Phase-contrast microscopy image (10× magnification) showing the typical fibroblast-like morphology of passage 3 murine BM-MSCs. **(B)** Flow cytometry confirming the expression of MSC-specific surface markers. Cells were positive for mesenchymal markers (CD44, CD73, CD90, CD106, and Sca-1) while negative for hematopoietic (CD34, CD11b) and MHC markers. Histograms display specific antibody staining (cyan) versus isotype controls (red). Representative of four experiments. **(C)** Gating strategy: lymphocytes identified by FSC/SSC, Doublets were excluded using FSC-H vs. FSC-A gating. viable NK cells defined as CD3⁻NKp46⁺, with dead cells excluded via Zombie staining. Further subdivision of NK cells into DX5⁻ and DX5⁺ subsets. Phenotypic analysis of NKG2A, NKG2D, Ly49A and Ly49C. Percentages indicate proportion within each gate. Data are analyzed using Student’s t-test, presented as mean ± SD(n=6/group). Data are representative of at least three independent experiments.

**Supplementary Figure 6. Phenotypic comparison of NKp46^+^DX5⁻ and NKp46^+^DX5⁺ liver NK cells under physiological and ACLF conditions**

**(A-D)** In healthy mice, NKG2A expression was significantly lower on DX5⁻ NK cells than DX5⁺ NK cell. No significant differences were observed in Ly49A, Ly49C or NKG2D. **(E-G)** In ACLF mice, MSC treatment increased Ly49A/C and decreased NKG2D on liver NK cells. %NKG2A^+^/%LY49A ^+^/ %LY49C^+^/% NKG2D^+^ indicates the proportion of positive cells among NKp46+DX5+/- liver mononuclear cells. Data are representative of at least three independent experiments. Data are analyzed using Student’s t-test, presented as mean ± SD (n=6/group). *** P<0.001.

**Supplementary Figure 7. DX5⁻ hepatic NK cells exhibit superior effector function upon strong stimulation**

(A–D) Cytokine levels in supernatants of sorted hepatic NKp46⁺DX5⁻ and NKp46⁺DX5⁺ NK cells from MSC or saline treated mice after PMA/ionomycin stimulation. DX5⁻ NK cells produced significantly higher levels of GM-CSF, IFN-γ, and IL-2 than DX5⁺ NK cells in both treatment groups. (E-H) qRT-PCR analysis of corresponding mRNA (*Csf2, Ifng, Il2, Tnf*) confirmed consistent upregulation in DX5⁻ NK cells. Data are analyzed using Student’s t-test, presented as mean ± SD (n = 12/group). * P<0.05, **p < 0.01, ***p < 0.001. Data are representative of at least three independent experiments.
