# Supplementary material for: Mesenchymal Stem Cells Ameliorate Acute‐on‐Chronic Liver Failure Associated With Expansion of Immunoregulatory Hepatic NKp46+DX5− NK Cells
Source: J Immunol Res. 2026 Sep 24;2026:2373331. doi: 10.1155/jimr/2373331 (PMC13602807; doi:10.1155/jimr/2373331)
Supplement: Supplementary file 1 [file JIMR-2026-2373331-s001.zip › Supplementary_File_1a.pdf]

華南農業大學
South China Agricultural University

动物实验伦理审查结果

编号：2024h052

实验项目名称	慢加急性肝衰竭小鼠模型的建立(国自然培育项目)
申请书编号	2024H052
实验动物保护评估意见	本实验所用实验小鼠来自有实验动物生产许可证单位。种类、数量及分组符合 3R 原则。
实验动物福利评估意见	本实验在有实验动物使用许可证的实验室进行，符合福利原则。
伦理道德评估意见	实验后的动物实施安乐死。
综合性科学评估意见	本实验研究有一定的科学意义。
实验时间、动物种类和数量	拟实验时间:2024 年 07 月 10 日至 2024 年 11 月 10 日，实验动物为 SPF 级 C57BL/6 小鼠 30 只（♂）
主任（或副主任）委员终审意见	同意。<br>审核人：[illegible]　审核时间：2024.7.9

华南农业大学实验动物伦理委员会

2024年 7 月 9 日

华南农业大学 实验动物伦理委员会
