# Supplementary material for: Mesenchymal Stem Cells Ameliorate Acute‐on‐Chronic Liver Failure Associated With Expansion of Immunoregulatory Hepatic NKp46+DX5− NK Cells
Source: J Immunol Res. 2026 Sep 24;2026:2373331. doi: 10.1155/jimr/2373331 (PMC13602807; doi:10.1155/jimr/2373331)
Supplement: Supplementary file 1 [file JIMR-2026-2373331-s001.zip › Supplementary_File_1b.pdf]

South China Agricultural University

Animal Experiment Ethics Review Results

No.: 2024h052

**Category**	**Details**
**Experimental Project Name**	Establishment of an acute-on-chronic liver failure mouse model (National Natural Science Foundation Incubation Project)
**Application No.**	2024H052
**Animal Protection Assessment Opinion**	The experimental mice used in this experiment are from a unit with an experimental animal production license. The species, quantity, and grouping comply with the 3R principles.
**Animal Welfare Assessment Opinion**	This experiment is conducted in a laboratory with an experimental animal use license, complying with welfare principles.
**Ethical Assessment Opinion**	Animals will be euthanized after the experiment.
**Comprehensive Scientific Assessment Opinion**	This experimental research has certain scientific significance.
**Experiment Period, Animal Species and Quantity**	Proposed experiment period: July 10, 2024 to November 10, 2024. Experimental animals are 30 SPF grade C57BL/6 mice(male).
**Final Review Opinion of the Director (or Deputy Director) of the Committee**	Approved.

Experimental Animal Ethics Committee of South China Agricultural University

July 09, 2024
