# Supplementary material for: Mesenchymal Stem Cells Ameliorate Acute‐on‐Chronic Liver Failure Associated With Expansion of Immunoregulatory Hepatic NKp46+DX5− NK Cells
Source: J Immunol Res. 2026 Sep 24;2026:2373331. doi: 10.1155/jimr/2373331 (PMC13602807; doi:10.1155/jimr/2373331)
Supplement: Supplementary file 1 [file JIMR-2026-2373331-s001.zip › Supplementary_File_1c.pdf]

附件 1

实验方案

一、实验动物（品种品系、性别、体重、数量、来源等）

品种品系： 4-6 周龄雄性 Balb/c 小鼠（SPF 级）；GFP 转基因 C57BL/6 小鼠（用于 MSCs 体内示踪）。均购自北京维通利华实验动物技术有限公司。

实验分组：小鼠随机分为以下 4 组（涉及 MSCs 注射的组别样本量为 n=10/组）：

对照组（Control）：注射等量橄榄油和生理盐水。

ACLF 模型组（CCL4 诱导）：仅进行 CCl_4造模。

ACLF + 生理盐水组（ACLF+saline）：CCl_4造模后给予生理盐水。

ACLF + MSCs 组（ACLF+MSC）：CCl_4造模后给予 MSCs 治疗。

二、实验周期

2024 年 07 月 10 日至 2024 年 11 月 10 日

三、实验过程（具体操作；进入实验室的试剂、用品；要在非实验室开放时间进行操作请说明）

模型建立与干预

- 慢性肝损伤：腹腔注射 10% CCl_4（使用橄榄油稀释，5 mL/kg），每周 2 次，持续 8 周。对照组注射等体积橄榄油。

- 急性失代偿：在第 8 周的第 3 天，单次腹腔注射亚致死剂量的 50% CCl_4（8 mL/kg）。
- MSCs 移植：健康的 Balb/c 小鼠或者 GFP 小鼠分离提取 BM-MSC，急性损伤后 1 小时，通过尾静脉注射 0.5×10^6个 MSCs（重悬于生理盐水中）或等体积生理盐水。
- 伦理措施：CCl_4剂量经预实验优化，确保亚致死性；密切监测小鼠精神状态、活动度、体重变化，出现严重痛苦（如体重下降>20%、无法进食）时立即实施安乐死。

样本采集与检测

- 时间点：急性 CCl_4处理后 0、6、12、24、48 小时。
- 血液采集：眼眶静脉丛采血，离心分离血清，检测 ALT/AST（Cobas 8000 分析仪）。
- 肝脏组织：4%多聚甲醛固定，石蜡包埋，5 μm 切片，H&E 染色评估病理损伤；BrdU（75 mg/kg，腹腔注射）标记增殖肝细胞，免疫组化检测。
- 伦理措施：采血前腹腔注射戊巴比妥钠（80 mg/kg）深度麻醉；组织采集在麻醉下进行，避免动物痛苦。

细胞实验

- MSCs 分离与鉴定：取 4-6 周龄 Balb/c 小鼠股骨/胫骨骨髓，密度梯度离心分离单个核细胞，贴壁培养传代，流式检测表面标志物。
- NK 细胞分离与功能分析：流式检测及流式分选。

- 伦理措施：细胞分离过程遵循无菌操作，减少动物使用数量（多只小鼠细胞混合以满足实验需求）；流式分选前活/死细胞染色排除死细胞，确保数据可靠性。

进入实验室的试剂包含戊巴比妥钠，4%多聚甲醛，物品包含小鼠手术器械，一次性注射器，小鼠固定器等。
