# Supplementary material for: Mesenchymal Stem Cells Ameliorate Acute‐on‐Chronic Liver Failure Associated With Expansion of Immunoregulatory Hepatic NKp46+DX5− NK Cells
Source: J Immunol Res. 2026 Sep 24;2026:2373331. doi: 10.1155/jimr/2373331 (PMC13602807; doi:10.1155/jimr/2373331)
Supplement: Supplementary file 1 [file JIMR-2026-2373331-s001.zip › Supplementary_File_1d.pdf]

Experimental Protocol

I. Experimental Animals (Species/Strain, Gender, Weight, Quantity, Source, etc.)

- **Species and Strain:** 4-6 week-old male Balb/c mice (SPF grade); GFP-transgenic C57BL/6 mice (used for in vivo tracking of MSCs). All were purchased from Beijing Vital River Laboratory Animal Technology Co., Ltd.
- **Experimental Grouping:** Mice are randomly divided into the following 4 groups (the sample size for groups involving MSCs injection is n=10/group):
- Control group: Injected with equivalent volumes of olive oil and normal saline.
- ACLF model group (CCL4-induced): Subjected to CCl_4 modeling only.
- ACLF + saline group (ACLF+saline): Administered normal saline after CCl_4 modeling.
- ACLF + MSCs group (ACLF+MSC): Treated with MSCs after CCl_4 modeling.

II. Experimental Period

July 10, 2024 to November 10, 2024

III. Experimental Procedures (Specific operations; reagents and supplies brought into the laboratory; please specify if operations need to be conducted outside of laboratory opening hours)

Model Establishment and Intervention

- **Chronic liver injury:** Intraperitoneal injection of 10% CCl_4 (diluted in olive oil, 5 mL/kg), twice a week for 8 weeks. The control group is injected with an equal volume of olive oil.
- **Acute decompensation:** On the 3rd day of the 8th week, a single intraperitoneal injection of a sub-lethal dose of 50% CCl_4 (8 mL/kg) is administered.
- **MSCs transplantation:** BM-MSCs are isolated and extracted from healthy Balb/c mice or GFP mice. One hour after the acute injury, 0.5×10^6 MSCs (resuspended in normal saline) or an equal volume of normal saline are injected via the tail vein.
- **Ethical measures:** The CCl_4 dose is optimized through preliminary experiments to ensure sub-lethality; the mice's mental state, activity level, and weight changes are closely monitored, and euthanasia will be performed immediately if severe distress occurs (e.g., weight loss >20%, inability to eat).

Sample Collection and Detection

- **Time points:** 0, 6, 12, 24, and 48 hours after the acute CCl_4 treatment.
- **Blood collection:** Blood is collected from the retro-orbital venous plexus, centrifuged to separate the serum, and tested for ALT/AST (Cobas 8000 analyzer).
- **Liver tissue:** Fixed in 4% paraformaldehyde, paraffin-embedded, sectioned at 5 μm, and subjected to H&E staining to evaluate pathological damage; BrdU (75 mg/kg, intraperitoneal injection) is used to label proliferating hepatocytes, which are then detected by immunohistochemistry.
- **Ethical measures:** Prior to blood collection, deep anesthesia is induced via intraperitoneal injection of sodium pentobarbital (80 mg/kg); tissue collection is performed under anesthesia to avoid animal suffering.

Cellular Experiments

- **MSCs isolation and identification:** Bone marrow from the femurs/tibias of 4-6 week-old Balb/c mice is collected, mononuclear cells are isolated via density gradient centrifugation, cultured adherently, passaged, and surface markers are detected

using flow cytometry.

- **NK cell isolation and functional analysis:** Flow cytometry detection and fluorescence-activated cell sorting (FACS).
- **Ethical measures:** The cell isolation process follows strict aseptic techniques to reduce the number of animals used (cells from multiple mice are pooled to meet experimental needs); live/dead cell staining is performed prior to flow sorting to exclude dead cells and ensure data reliability.

Reagents and Supplies Brought into the Laboratory

Reagents include sodium pentobarbital and 4% paraformaldehyde, and supplies include mouse surgical instruments, disposable syringes, mouse restrainers, etc.
