# Supplementary material for: Mesenchymal Stem Cells Ameliorate Acute‐on‐Chronic Liver Failure Associated With Expansion of Immunoregulatory Hepatic NKp46+DX5− NK Cells
Source: J Immunol Res. 2026 Sep 24;2026:2373331. doi: 10.1155/jimr/2373331 (PMC13602807; doi:10.1155/jimr/2373331)
Supplement: Supplementary file 1 [file JIMR-2026-2373331-s001.zip › Supplementary_File_1e.pdf]

文件编号：SL-YW2023-004-02

同意函

同意函号：中大附三医伦 YW2023-004-02　　　　同意日期：2023-01-31

项目名称	一项评价 CG-BM1 异体人骨髓间充质干细胞注射液治疗慢加急性肝衰竭患者的安全性、耐受性和初步有效性临床试验
项目负责人	林炳亮
项目负责人科室	感染性疾病科
申办方	天津凯诺医药科技发展有限公司
审查类别	初始审查、复审
审查方式	会议审查、快速审查
同意使用文件	1.修改后的知情同意书（版本号：V1.2, 版本日期：2023-01-16）<br>2.临床研究方案（版本号：V1.1, 版本日期：2023-01-04）<br>3.招募广告（版本号：V1.0, 版本日期：2022-12-15）<br>4.受试者日记卡（版本号：V1.0, 版本日期：2022-12-15）
审查意见	本伦理委员会（参加委员名单见附件）于 2023 年 01 月 11 日召开会议，严格按照 ICH-GCP 原则及中国相关的法规/指南，审阅并讨论了该项目。应到人数 17 人，其中因事请假 4 人，实到人数 13 人，回避人数 0 人。投票结果：2 位委员表示“同意”，11 位委员表示“必要的修改后同意（快审）”。<br>审查结果：必要的修改后同意（快审）<br>具体意见如下：<br>一、知情同意书<br>1.关于损害赔偿不应当增加需要权威部门鉴定的限制，建议删除“经国家法律法规规定的权威机构认定需要承担相应责任的”的字样。<br>2.明确女性受试者避免怀孕的具体时间。<br><br>请根据意见尽快修改后提交“复审”申请。<br>如果对审查意见有不同观点，可以“复审申请”的方式申诉不同意见，请伦理委员会重新考虑决定。再次送审时请递交以下文件：<br>（1）送审文件清单<br>（2）修订记录（列表说明）<br>（3）修改后的研究方案（含新版本号），对修改部分用醒目字体标出。<br>（4）修改后的知情同意书（含新版本号），对修改部分用醒目字体标出。<br>（5）其他需要补充的文件。<br>2023 年 01 月 18 日研究者递交伦理意见回复本伦理委员会于 2023 年 01 月 31 日严格按照 ICH-GCP 原则及中国相关的法规/指南，由 2 位委员通过快审形式审阅了该项目。审查结果：同意
年度/定期跟踪审查频率	12 个月，截止日期：2024 年 01 月 30 日
同意函失效日期	2024 年 01 月 30 日

地址：广东省广州市天河路 600 号　　邮编：510630　　电话：020-85252621

主任委员或副主任委员签名：

中山大学附属第三医院医学伦理委员会（盖章）
日期：2023 年 01 月 31 日

注意事项：

1. 请遵循 NMPA/GCP，《药物临床试验伦理审查工作指导原则》和《赫尔辛基宣言》的原则、遵循伦理委员会批准的方案开展临床研究，保护受试者的健康与权利。
2. 研究过程中若变更主要研究者，对临床研究方案、知情同意书、招募材料等的任何修改，请申请人提交修正案审查申请。
3. 发生 SAE/SUSAR，请申请人及时提交。
4. 请按照伦理委员会规定的年度定期跟踪审查频率，申请人在截止日期前 1 个月提交年度定期跟踪审查报告。
5. 申请人暂停或提前终止临床研究，请及时提交暂停/终止研究报告。
6. 完成临床研究，请申请人提交结题报告。

声明：

研究实施前提：

1.所有研究需经伦理委员会审查获得同意函后方可实施。

2.如属《人类遗传资源采集、收集、买卖、出口、处境审批行政许可事项》规定范畴的研究，需获得中国人类遗传资源管理部门的批准/备案成功后方可实施。

附：审阅文件目录：

2023-01-06 初始审查审阅目录如下：

1. 药物临床试验伦理初始审查申请
2. 研究经济利益声明
3. 中山大学附属第三医院药物临床试验申请表
4. NMPA 临床试验批件
5. NMPA 受理通知书
6. 参与试验的国家、国内中心及相应计划入组例数
7. 方案摘要（版本号：V1.1，版本日期：2023-01-04）
8. 知情同意书（版本号：V1.1，版本日期：2023-01-05）
9. 研究者手册（版本号：V1.0，版本日期：2022-12-15）
10. 病例报告表（版本号：0.1，版本日期：2022-12-16）
11. 保险声明
12. 主要研究者的资格证明文件
13. 申办者对所提交资料真实性声明
14. CG-BM1 药检报告
15. 复方电解质注射液药检报告
16. 研究团队成员表
17. 药物临床试验伦理初始审查申请签字扫描版

2023-01-20 复审审阅目录如下：

1. 复审申请
2. 知情同意书修订记录
3. 复审申请表签字扫描版

地址：广东省广州市天河路 600 号　邮编：510630　电话：020-85252621
