# Supplementary material for: Mesenchymal Stem Cells Ameliorate Acute‐on‐Chronic Liver Failure Associated With Expansion of Immunoregulatory Hepatic NKp46+DX5− NK Cells
Source: J Immunol Res. 2026 Sep 24;2026:2373331. doi: 10.1155/jimr/2373331 (PMC13602807; doi:10.1155/jimr/2373331)
Supplement: Supplementary file 1 [file JIMR-2026-2373331-s001.zip › Supplementary_File_1f.pdf]

Medical Ethics Committee of the Third Affiliated Hospital of Sun Yat-sen University
Document No.: SL-YW2023-004-02

Letter of Approval

Approval Letter No.: The Third Affiliated Hospital of Sun Yat-sen University YW2023-004-02
Approval Date: 2023-01-31

**Project Name**	A clinical trial to evaluate the safety, tolerability, and preliminary efficacy of CG-BM1 allogeneic human bone marrow mesenchymal stem cell injection in the treatment of patients with acute-on-chronic liver failure.
**Project Investigator**	Lin Bingliang
**Department of PI**	Department of Infectious Diseases
**Sponsor**	Tianjin Kai Nuo Medical Technology Development Co., Ltd.
**Review Category**	Initial Review, Re-review
**Review Method**	Meeting Review, Expedited Review
**Approved Documents for Use**	1. Modified Informed Consent Form (Version: V1.2, Version Date: 2023-01-16)<br>2. Clinical Research Protocol (Version: V1.1, Version Date: 2023-01-04)<br>3. Recruitment Advertisement (Version: V1.0, Version Date: 2022-12-15)<br>4. Subject Diary Card (Version: V1.0, Version Date: 2022-12-15)
**Review Opinions**	This Ethics Committee (see appendix for the list of participating members) convened a meeting on January 11, 2023. In strict accordance with ICH-GCP principles and relevant Chinese laws/guidelines, the project was reviewed and discussed. The expected number of attendees was 17; 4 members asked for leave due to personal reasons, and the actual number of attendees was 13, with 0 members needing to recuse themselves. Voting results: 2 members voted "Approve", 11 members voted "Approve after necessary modifications (expedited review)".

	Review Result: Approve after necessary modifications (expedited review).<br><br>Specific opinions are as follows:<br>**I. Informed Consent Form**<br>1. Regarding compensation for damages, the restriction requiring appraisal by authoritative departments should not be added. It is recommended to delete the phrase "which needs to bear corresponding responsibility as confirmed by authoritative institutions as stipulated by national laws and regulations."<br>2. Clarify the specific timeframe for female subjects to avoid pregnancy.<br><br>Please make modifications according to the opinions and submit an application for "re-review" as soon as possible.<br>If you disagree with the review opinions, you may appeal by submitting an application for "re-review" to ask the Ethics Committee to reconsider the decision. When submitting for review again, please submit the following documents:<br>(1) List of submitted documents<br>(2) Record of modifications (list explanation)<br>(3) Modified research protocol (including new version number), with modified parts highlighted in conspicuous fonts.<br>(4) Modified informed consent form (including new version number), with modified parts highlighted in conspicuous fonts.<br>(5) Other documents requiring supplementation.<br><br>On January 18, 2023, the researcher submitted the responses to the ethics opinions to the Ethics Committee. On January 31, 2023, in strict accordance with ICH-GCP principles and relevant Chinese laws/guidelines, 2 committee members reviewed the project via expedited review.<br>Review result: Approved.
**Annual/Periodic Follow-up Review Frequency**	12 months, Deadline: January 30, 2024
**Expiration Date of Approval Letter**	January 30, 2024

Page 2: Signatures and Appendix

Signature of Chairperson or Deputy Chairperson: *Xie dongying*

(Seal): Medical Ethics Committee of the Third Affiliated Hospital of Sun Yat-sen University

Date: January 31, 2023

Precautions:

1. Please conduct the clinical research and protect the health and rights of the subjects in compliance with NMPA/GCP, the *Guiding Principles for Ethical Review of Drug Clinical Trials*, the principles of the *Declaration of Helsinki*, and the protocol approved by the Ethics Committee.
2. If there is a change in the principal investigator during the research process, or any modifications to the clinical research protocol, informed consent form, recruitment materials, etc., the applicant must submit an amendment review application.
3. Upon the occurrence of an SAE/SUSAR, the applicant is requested to submit it in a timely manner.
4. Please follow the annual periodic follow-up review frequency stipulated by the Ethics Committee; the applicant must submit the annual periodic follow-up review report 1 month before the deadline.
5. If the applicant suspends or terminates the clinical research early, please submit a suspension/termination report in a timely manner.
6. Upon completion of the clinical research, the applicant is requested to submit a conclusion report.

Statement:

Prerequisites for research implementation:

1. All research can only be implemented after obtaining the letter of approval from the Ethics Committee review.
2. If it falls within the scope of research stipulated in the *Administrative Licensing Items for the Collection, Gathering, Trading, Export, and Processing Approval of Human Genetic Resources*, it can only be implemented after successfully obtaining approval/filing from the human genetic resources management department.

Appendix: List of Reviewed Documents:

2023-01-06 Initial Review Document List:

1. Application for initial ethical review of drug clinical trials
2. Statement of economic interest in research
3. Application form for drug clinical trials at the Third Affiliated Hospital of Sun Yat-sen University
4. NMPA Clinical Trial Approval Document
5. NMPA Acceptance Notice
6. National and domestic centers participating in the trial and corresponding planned number of enrolled cases
7. Protocol Synopsis (Version: V1.1, Version Date: 2023-01-04)
8. Informed Consent Form (Version: V1.1, Version Date: 2023-01-05)
9. Investigator's Brochure (Version: V1.0, Version Date: 2022-12-15)
10. Case Report Form (Version: 0.1, Version Date: 2022-12-16)
11. Insurance Statement

12. Principal investigator's qualification documents
13. Sponsor's statement on the authenticity of submitted materials
14. CG-BM1 drug inspection report
15. Compound electrolyte injection drug inspection report
16. List of research team members
17. Scanned copy of the signature for the initial ethical review application of the drug clinical trial

2023-01-20 Re-review Document List:

1. Application for re-review
2. Record of modifications to the informed consent form
3. Scanned copy of the signature for the re-review application

Address: No. 600 Tianhe Road, Guangzhou, Guangdong Province **Zip Code:** 510630 **Phone:** 020-85252621
