# Supplementary material for: Mesenchymal Stem Cells Ameliorate Acute‐on‐Chronic Liver Failure Associated With Expansion of Immunoregulatory Hepatic NKp46+DX5− NK Cells
Source: J Immunol Res. 2026 Sep 24;2026:2373331. doi: 10.1155/jimr/2373331 (PMC13602807; doi:10.1155/jimr/2373331)
Supplement: Supplementary file 1 [file JIMR-2026-2373331-s001.zip › Supplementary_File_1g.pdf]

评价异体人骨髓间充质干细胞注射液治疗慢加急性肝衰竭患者的安全性、耐受性和初步有效性临床试验

1. 背景

乙型肝炎病毒引起的慢加急性肝衰竭（ACLF）死亡率很高。目前唯一有效的治疗方法是原位肝移植。然而，该疗法的应用受限于供体器官短缺、排斥反应以及移植后免疫抑制药物的大量使用。因此，临床上迫切需要其他有效、安全且方便的治疗方法。

骨髓间充质干细胞（BM-MSCs）具有自我更新和多向分化的特性。研究表明，BM-MSCs 具有归巢至受损肝脏的能力，可分化为肝细胞，抑制肝纤维化的进展，并且体外研究未显示致瘤性。在终末期肝病患者的临床实践中，自体 BM-MSCs 移植安全且无排斥反应，可改善肝功能。然而，关于 BM-MSCs 移植后长期疗效的研究仍有限。

由于 BM-MSCs 不表达人白细胞抗原（HLA）II 类分子或共刺激分子，并且具有免疫抑制特性，我们假设对乙肝相关慢加急性肝衰竭患者输注异体 MSCs 可能是安全的，不会引起排斥反应，并能改善预后。

2. 研究设计

本研究为单中心、II 期、前瞻性、开放标签、随机对照临床试验。

3. 研究目的

评估异体 BM-MSCs 输注治疗乙肝相关慢加急性肝衰竭患者的安全性和有效性。此外，对患者外周血淋巴细胞的免疫学特征进行初步研究。

4. 患者与知情同意

由于是单中心临床试验，我们重点关注确诊为乙肝相关慢加急性肝衰竭的中国患者。对于符合入选标准的患者，需取得患者本人或其亲属/监护人的书面知情同意。

5. 入选标准

a. 符合亚太肝脏研究学会（APASL）2009 年共识建议的慢加急性肝衰竭（ACLF）患者；
b. 血清乙肝表面抗原（HBsAg）阳性超过 6 个月；
c. 终末期肝病模型（MELD）评分范围为 17-30 分；
d. 年龄范围 16-60 岁。

6. 排除标准

a. 2 个月内存在严重并发症（例如：消化道出血、严重感染）等特殊情况的患者；
b. 合并自身免疫性疾病；
c. 合并感染其他肝炎病毒；
d. 存在重要器官功能障碍（例如肾功能障碍：血清肌酐水平>116 µmol/L 或尿蛋白阳性）或恶性肿瘤；
e. 妊娠期及哺乳期；
f. 过度饮酒；
g. 6 个月内使用过肝毒性药物；
h. 经超声、计算机断层扫描（CT）或磁共振（MR）证实存在的肝肿瘤或肝硬化结节；
i. 接受生物人工肝支持治疗或肝移植。

7. 样本量
样本量的确定基于以下标准：显著性水平 α = 0.05（双侧）；检验效能 = 0.8；对照组比例 = 0.50；基于前期研究，对照组死亡率 = 54.7%；^26^ 预估治疗获益 = 50%。计算得出样本量为 102 例（每组 51 例）。考虑约 10%的脱落或失访率，本研究最终入组样本量为 112 例患者。

8. 随机化与设盲
我们按 1：1 比例将患者随机分配至接受标准药物治疗组（SMT 组）或 BM-MSCs 输注组（MSC 组）。患者通过计算机生成的随机化序列进行随机分配。生成随机化序列的统计人员不参与研究的其余部分。本研究为开放标签研究。参与者和研究团队均知晓治疗分组情况。

9. 治疗方案与随访
a. 预计入组 112 名受试者，由中山大学统计部门按 1：1 比例分配至异体 BM-MSCs 组（MSC 组，56 名受试者）或标准药物治疗组（SMT 组，56 名受试者）。

b. 所有患者均接受标准药物治疗，包括卧床休息、补充能量、人血白蛋白和血浆、恩替卡韦以及并发症治疗。MSC 组在标准药物治疗基础上，每周一次经外周静脉输注异体 BM-MSCs，共 4 次。BM-MSCs 输注量根据体重调整，为 5.0×10^{5}/Kg。

c. 观察与随访时间点为基线（首次输注前）、首次输注后第 1、2、3、4、8、12 和 24 周。

d. 在获得知情同意后，采集健康对照者和不同时间点患者的外周血样本。分离外周血单个核细胞（PBMC），通过流式细胞术检测 NK 细胞百分比、抑制性 NK 受体和激活性 NK 受体的表达比例。

10. 相关定义
a. 入组：患者入院时即进行筛选，符合入选标准者将被告知关于慢加急性肝衰竭新疗法的详细信息。签署书面知情同意后，患者将获得病例报告表（CRF）编号。

b. 退出：患者可随时无理由退出研究，其个人及临床信息将被保密。

c. 基线：一旦入组，患者将被随机分配至不同组别。对于接受 MSC 治疗的患者，将首次 MSC 输注前一天定义为基线。对于接受标准药物治疗的患者，将患者入组研究当天定义为基线。

d. 随访：我们将在每个时间点进行随访，包括基线、第 1、2、3、4、8、12 和 24 周。对于接受 MSC 输注的患者，我们在每个时间点（第 1-4 周）的 MSC 输注前收集数据。患者出院后，我们为其建立随访系统，提前通知患者回院复查。若患者因某些原因（如病情恶化）无法及时返回，我们将要求其在当地医院进行肝功能检查，并通过电话收集数据。

e. 研究治疗的永久终止
研究治疗永久终止的患者（以下各类别的人数和百分比）；
撤回知情同意的患者；
因严重不良事件而终止研究治疗；

因治疗重点转为肝移植或生物人工肝支持治疗而终止研究治疗；
因其他原因终止研究治疗。

11. 安全性指标定义

发热、皮疹、腹泻、肺栓塞和肝细胞癌可能是异体 MSC 输注后的主要不良反应。无论是否与 MSC 输注相关，我们都将在随机后观察和记录上述不良反应，包括发生时间、持续时间及结局。
发热：腋下体温大于 37.3℃。
皮疹：皮肤出现点状疹。
腹泻：过量且频繁（每日超过 3 次）排出水样便。
肺栓塞：表现为胸痛、咯血和呼吸困难，并经胸部影像学结果证实。
肝细胞癌：经超声、计算机断层扫描（CT）或磁共振（MR）证实的肝癌。

12. 疗效指标定义

12.1 主要终点指标定义
主要终点指标为随机化后 24 周生存率和输注后不良反应发生率。
我们记录每个时间点患者的生存状态。例如，若患者在随机化后第 3 周至第 4 周之间死亡，我们将记录其生存时间为 3 周。对于未回院复查的患者，我们将通过电话获取其生存状态。不良反应的定义见“安全性指标”部分。

12.2 次要终点指标定义
次要终点指标包括：
(1) 随机化后对肝功能（例如：丙氨酸氨基转移酶（ALT）、白蛋白（ALB）、总胆红素（TBil）、国际标准化比值（INR）、终末期肝病模型（MELD）评分）的影响；
(2) 随机化后致死性并发症（例如：感染、肝性脑病、肝肾综合征、消化道出血、中毒性肠麻痹）的发生率。

12.3 第三终点指标定义
第三终点指标包括：
(1) 各时间点白细胞计数（WBC）、血红蛋白（Hb）、血小板（PLT）和肌酐（Cr）的变化；
(2) 肝脏大小和腹水的变化（数据由各时间点的超声检查提供）；
(3) 各时间点 IgM、IgG、IgE、C3、C4、CH50 水平的变化。

13. 分析原则

(1) 所有分析均基于意向性治疗（ITT）原则进行。
(2) 所有分析均由专业统计人员执行。
(3) 所有检验均为双侧检验，名义显著性水平α设定为 5%。
(4) 除特别说明外，所有统计分析均未经调整。
(5) P 值不因多重性进行调整。但结果按重要程度（主要至第三）明确分类。

14. 中期分析

独立数据监测委员会（DMC）将在一次中期分析（获得 56 名患者的主要结局数据时）和最终分析时，审查患者特征、治疗依从性和研究结局的非盲态数据。试验期间将定期审查入组情况，并制定相关职责范围。DMC 的职责是，如果在任何时候出现超出合理怀疑范围的随

机分组间死亡率差异的证据，或出现可能改变已熟悉现有试验干预措施证据的许多临床医生实践的迹象，则需通知研究管理和执行委员会。

15. 统计分析

15.1 试验流程

将通过 CONSORT 流程图展示患者在研究中的流动情况。我们将报告符合研究入选标准的筛选患者数量、纳入患者数量、未纳入患者的排除原因及相关信息，如附录 I 所示。

15.2 患者特征与基线比较

a. 将针对一系列基线变量生成汇总统计表：年龄（连续变量）、性别、白细胞计数（连续变量）、血红蛋白（连续变量）、血小板（连续变量）、肌酐（连续变量）、肝功能指标（如丙氨酸氨基转移酶、白蛋白、总胆红素、国际标准化比值、终末期肝病模型评分）（连续变量）、HBV-DNA 滴度（连续变量）、HBeAg 状态、是否合并肝硬化、ACLF 患者的并发症（如感染、肝性脑病、肝肾综合征、消化道出血、中毒性肠麻痹）。附录 II 展示了将生成的表格模板。

b. 连续变量将使用以下统计量进行汇总：样本量（非缺失样本量）、均值、标准差、中位数和四分位距（IQR）。所有分类变量将报告观察水平的频率和百分比（基于非缺失样本量）。将采用标准 X^2检验比较频率，连续数据采用 t 检验或 Wilcoxon 秩和检验。对于罕见事件（预期每格计数小于 1），将采用 Fisher 精确检验。

15.3 主要终点指标（包括安全性结局）分析

a. 主要分析将遵循意向性治疗（ITT）原则，比较随机分组间 24 周（全因）死亡率和不良反应发生率。我们将制作表格记录每个时间点患者的生存状态（附录 III），并计算生存率。绘制 Kaplan-Meier 曲线，并使用 Log-rank 检验进行比较。对于失访患者，我们在分析中将不进行缺失数据填补。

b. 我们将记录每个时间点的不良反应，并使用卡方检验比较两组在最初 4 周和最后 20 周内不良反应的发生频率。

15.4 次要终点指标分析

a. 次要分析将比较 MSC 组与 SMT 组之间的肝功能（如 ALT、ALB、TBil、INR、MELD 评分）和致死性并发症（如感染、肝性脑病、肝肾综合征、消化道出血、中毒性肠麻痹）的发生率。我们将记录每个时间点的肝功能数据和并发症情况。

b. 我们将比较两个时间段（例如最初 4 周和整个 24 周）的肝功能指标。dALT、dALB、dTBil、dINR 和 dMELD 评分定义为基线至相应时间段内最佳值之间的差值（D 值），并使用 t 检验比较这些变量。

c. 我们将使用标准 X^2检验比较 MSC 组与 SMT 组在整个随访期间致死性并发症的发生率。对于罕见事件（预期每格计数小于 1），将采用 Fisher 精确检验。

15.5 第三终点指标分析

a. 第三分析将比较各时间点的 WBC、Hb、PLT、Cr、肝脏大小、腹水以及 IgM、IgG、IgE、C3、C4、CH50 水平。除腹水外，将使用*t*检验或 Wilcoxon 秩和检验。

b. 我们对经超声检测的腹水进行分级定义：

0 = 无;

1 = 轻度;

2 = 中度;

3 = 大量。
组间比较将使用标准 X^2检验或 Fisher 精确检验。

15.6 安全性终点分析
安全性终点分析将包括不良反应（第 15.3 节）以及 WBC、Hb、PLT 和 Cr（第 15.5 节）的比较。

16. 缺失数据处理
主要结局指标分析应尽量无缺失数据。将尽一切努力确保至少获取每位患者 24 周死亡率的完整信息。
其他结局指标或协变量中出现的缺失数据将在主要分析中进行多重填补, 以提高估计值的精确度并避免完全案例分析可能带来的偏倚。将进行敏感性分析以评估多重填补的影响，同时也会进行完全案例分析。将检查所有填补值以确保生成合理的数值。填补模型将包含基线变量和结局变量。
