# Supplementary material for: Mesenchymal Stem Cells Ameliorate Acute‐on‐Chronic Liver Failure Associated With Expansion of Immunoregulatory Hepatic NKp46+DX5− NK Cells
Source: J Immunol Res. 2026 Sep 24;2026:2373331. doi: 10.1155/jimr/2373331 (PMC13602807; doi:10.1155/jimr/2373331)
Supplement: Supplementary file 1 [file JIMR-2026-2373331-s001.zip › Supplementary_File_1h.pdf]

A Clinical Trial to Evaluate the Safety, Tolerability and Preliminary Efficacy of Allogeneic Human Bone Marrow-Derived Mesenchymal Stem Cell Injection in Patients with Acute-on-Chronic Liver Failure

1. Background

Acute-on-chronic liver failure (ACLF) caused by hepatitis B has a high mortality. The only effective therapy for it is orthotopic liver transplantation. However, the applicability of this therapy is limited by the shortage of donor organs, rejection, and the massive use of immunosuppressive medicine after transplantation. Therefore, other effective, safe, and convenient therapeutic approaches are needed urgently.

Bone marrow-derived mesenchymal stromal cells (BM-MSCs) are featured by the characteristics of self-renewal and multipotentiality. Researches showed that BM-MSCs possess the ability of homing to the damaged liver, differentiating into hepatocytes, inhibiting the progress of liver fibrosis, and having no tumorigenicity *in vitro*. In the clinical practice for end-stage liver disease patients, autologous BM-MSCs transplantation was safe without rejection response, which improved the liver function. However, the long-term efficacy of BM-MSCs transplantation was limited.

Because BM-MSCs do not express human leukocyte antigen (HLA) class II or co-stimulatory molecules and have immunosuppressive properties, we hypothesized that allogeneic MSCs infusion for HBV-related ACLF patients may be safe without rejection reactions and improve the outcomes.

2. Study design

This study is a single center, phase 2, prospective, open label, randomised controlled clinical trial.

3. Objective

To evaluate the safety and efficacy of allogeneic BM-MSCs infusion in the treatment of patients with HBV-related acute-on-chronic liver failure. Furthermore, a preliminary study on the immunological characterization of peripheral blood lymphocytes.

4. Patient population and consent

As it is a single center clinical trial, we pay our attention to the Chinese people who are diagnosis with HBV-related acute-on-chronic liver failure. For patients who meet the eligibility criteria, written informed consent is taken from either the patient or a relative/guardian/carer.

5. Inclusion criteria：

a**.** Acute-on-chronic liver failure (ACLF) patients according to consensus recommendations of APASL 2009.

b. Positive serum hepatitis B surface antigen (HBsAg) more than 6 months;

c. Model for End-Stage Liver Disease (MELD) score range 17-30;

d. Age span 16-60 years.

.

6. Exclusion criteria:

a. Patients had some special conditions such as serious complications within 2 months (e.g., gastrointestinal bleeding, serious infection);
b. Concomitant autoimmune diseases;
c. Superinfection with other hepatitis viruses;
d. Suffer important organ dysfunctions (e.g. kidney dysfunction: patients with serum creatinine level >116umol/L or urinary protein positive) or malignancies;
e. Pregnancy and lactation;
f. Excessive alcohol drinking;
g. Intaking hepatotoxic drugs within 6 months;
h. Liver tumor or nodular secondary to cirrhosis proven by ultrasound, computerized tomography (CT), or magnetic resonance (MR);
i. Receiving bioartificial liver support therapy or liver transplantation.

7. Sample size

To decide for the sample size, the following criteria are taken into consideration: level = 0.05 (2-side); statistical power = 0.8; proportion in control group = 0.50; mortality in control group = 54.7%, based on the prior research; and the estimated treatment benefit = 50%. The sample size is 102 (51 in each group). Considering about 10 percent dropouts or loss to follow-up, this study final enrolled samples are 112 patients.

8. Randomization and masking

We randomly allocated patients in a 1:1 ratio to receive the standard medicine treatment (Group SMT) or BM-MSCs infusions (Group MSC). Patients were randomly allocated with a computer-generated randomization sequence. The statistician who generated the randomization sequence was not involved in the rest of the study. This is an open-label study. Both participants and the study team were unmasked to treatment allocation.

9. Therapeutic regimen and follow up

a. 112 participants are enrolled anticipatively, and allocated in 1:1 into allogeneic BM-MSCs (MSC group, 56 participants) or standard medicine therapy (SMT group, 56 participants) by the department of statistics of Sun Yat-sen University. （See Randomization section）

b. All the patients received the standard medicine treatment, including bed rest, supplement of energy, human serum albumin and plasma, entecavir and the treatment for complications. Group MSC took four doses of allogeneic BM-MSCs infusions via peripheral veins once a week, in addition to the standard medicine treatment. The infusion amount of BM-MSCs, adjusted by body weight, was 5.0×10^5/Kg.

c. Observation and follow-up were performed from the beginning, 1, 2, 3, 4, 8, 12 and 24 weeks after the first infusion.

d. Blood samples were collected with informed consent from healthy controls, patients in different time point. Peripheral blood mononuclear cells (PBMC) were isolated, and percentage of natural killer cell, inhibitory NK receptors and activating NK receptors were determined by flow cytometry analysis.

10. Definition of some issues

a. Enrollment: We screen patients once they are admitted to hospital, and those who meet the eligibility criteria will be informed the new therapy for acute-on-chronic liver failure in detail. After written consent signing, the patient will get a CRF (Case Report Form) number.

b. Withdraw: Patients can withdraw from the study at any time for no reasons and their private and clinical information will be kept secret.

c. Baseline: Once enrolled in the study, patients will be allocated into different groups randomly. For those who receive MSC treatment, we define the day before first time MSC infusion as the baseline. And for those who receive standard medicine treatment, we define the day patients enroll in the study as the baseline.

d. Follow-up: We will perform follow-up at each time point, including baseline, 1, 2, 3, 4, 8, 12 and 24 weeks. For patients who receive MSC infusion, we collect data at each time point (from 1-4 weeks) before MSC infusion. After patients discharging from hospital, we set up a follow-up system for them. Patients will be informed to come back to the hospital in advance. If patients could not return in time for some reasons (e.g. patient's condition get worse), we will ask them to have liver function test at local hospital and collect the data by telephone.

e. Permanent discontinuation of study treatment

Patients for whom study treatment is permanently discontinued (number and % in each of the following categories);

Patients for whom informed consent is withdrawn;

Study treatment discontinued because of serious adverse event;

Study treatment discontinued as focus of treatment changed to liver transplantation or bioartificial liver support therapy;

Study treatment discontinued for other reasons.

11. Definition of safety variables

Fever, rash, diarrhea, pulmonary embolism and hepatocellular carcinoma (HCC) may be the main adverse reactions after allogeniec MSC infusion. We observe and record the adverse reactions mentioned above after randomisation, including the onset time, duration and the outcomes, no matter whether they are related to the MSC infusion or not.

Fever: Axillary temperature greater than 37.3°C.

Rash: An eruption of spots on the skin.

Diarrhea: Excessive and frequent (more than 3 times daily) evacuation of watery feces.

Pulmonary embolism: Characterized by chest pain, hemoptysis and dyspnea, confirmed by chest radiograph results.

Hepatocellular carcinoma: Liver cancer proven by ultrasound, computerized tomography (CT), or magnetic resonance (MR).

12. Definition of efficacy variables

12.1 Definition of primary endpoints

The primary end points are the 24-week survival rate after randomization and the incidence of adverse reactions after infusion.

We record the survival status of patients at each time point. For example, if a patient die between week 3 and 4, we will record the patient's survival time is 3 weeks. For those patients who do not return to our hospital, we will get their survival status by telephone.

Adverse reactions are defined in the "safety variables" section.

12.2 Definition of secondary endpoints

The secondary end points will include:

(1) Influence on liver function (e.g. alanine aminotransferase (ALT), albumin (ALB), total bilirubin (TBil), international normalize ratio (INR), model for end stage liver disease (MELD) scores) after randomisation;

(2) Incidence of fatal complications (e.g. infection, encephalopathy, hepatorenal syndrome, gastrointestinal bleeding, toxic enteroparalysis) after randomisation;

12.3 Definition of tertiary endpoints

The tertiary end points will include:

(1) Changes of white blood count (WBC), hemoglobin (Hb), platelets (PLT) and creatinine (Cr) at each time point;

(2) Changes of liver size and ascites.(the data will be provided by ultrasound at each time point)

(3) Changes of levels of IgM, IgG, IgE, C3, C4, CH50 at each time point;

13. Analysis principles

(1) All analyses will be conducted on an intention-to-treat basis.

(2) All analysis will be performed by professional statisticians.

(3) All tests will be two-sided, and the nominal level of α will be 5%.

(4) All statistical analyses will be unadjusted except where indicated.

(5) *P* values will not be adjusted for multiplicity. However, the outcomes are clearly categorised by degree of importance (primary to tertiary).

14 Interim analyses

An independent Data Monitoring Committee (DMC) will review unblinded data on patient characteristics, treatment compliance and study outcomes at one interim analyses (availability of primary outcome for 56 patients), and at the final analysis. Recruitment will be reviewed at regular intervals during the trial, which will generate terms of reference. The DMC is charged with informing the Study Management and Executive Committees if at any time there emerges either evidence beyond reasonable doubt of a difference between randomized groups in mortality, or evidence likely to change the practice of many clinicians already familiar with the available evidence about the trial interventions.

15. Statistical analysis

15.1 Trial profile

Flow of patients through the study will be displayed in a CONSORT diagram. We will report the

number of screened patients who met study inclusion criteria and the number included, reasons for exclusion of non-included patients and information.

15.2 Characteristics of patients and baseline comparisons

a. Table of summary statistics will be produced for a number of baseline variables; age (continuous), sex, blood white blood cell (continuous), hemoglobin (continuous), platelet (continuous), creatinine (continuous), liver function(e.g. alanine aminotransferase, albumin , total bilirubin, international normalize ratio , model for end stage liver disease scores) (continuous), HBV-DNA titers (continuous), HBeAg status, whether suffer cirrhosis, complications of ACLF patients (e.g. infection, encephalopathy, hepatorenal syndrome, gastrointestinal bleeding, toxic enteroparalysis).

b. Continuous variables will be summarised using the following statistics; n (non-missing sample size), mean, standard deviation, median and inter-quartile range (IQR). The frequency and percentages (based on the non-missing sample size) of observed levels will be reported for all categorical measures. Standard X^2 tests will be performed to compare frequencies, and *t* tests or Wilcoxon rank sum tests will be used for continuous data. In case of rare events (expected number per cell less than 1), the Fisher test will be used.

15.3 Primary end points (including safety outcomes) analysis

a. The primary analysis will be a comparison of 24-week (all cause) mortality and the incidence of adverse reactions between the randomized groups, following an intent-to-treat (ITT) policy. We will produce a table to record the patients' survival status at each time point. Survival rates will be calculated. The Kaplan-Meier curve will be plotted, which is compared using Log-rank test. For those patients who are lost to follow-up, we will not input the missing data for analysis.

b. We will record adverse reactions at each time point, and the frequency of the reactions between groups will be compared during the first four weeks and last 20 weeks using Chi-square test.

15.4 Secondary end points analysis

a. The secondary analysis will be a comparison of liver function (e.g. ALT, ALB, TBil, INR, MELD scores) and the incidence of fatal complications (e.g. infection, encephalopathy, hepatorenal syndrome, gastrointestinal bleeding, toxic enteroparalysis) between MSC Group and SMT Group. We will record the data of liver function and complications at each time point;

b. We compare the liver function in 2 periods (e.g. the first 4 weeks and the whole 24 weeks). dALT, dALB, dTBil, dINR and dMELD score are defined as the D-value of liver function between baseline and the most favorable value in corresponding period. And the variables are compared using *t* test.

c. We will compare the incidence of fatal complications between MSC Group and SMT Group during the whole period of follow-up using standard X^2 tests. In case of rare events (expected number per cell less than 1), the Fisher test will be used.

15.5 Tertiary end points analysis

a. The tertiary analysis will be a comparison of WBC, Hb, PLT, Cr, liver size, ascites and levels of IgM, IgG, IgE, C3, C4, CH50 at each time point. And *t* tests or Wilcoxon rank sum tests will be used except for ascites.

b. We define different categories for the ascites, which will be detected by ultrasound.

0=none;
1=mild;
2=moderate;
3=large.

And comparison between groups will use standard X^2 tests or Fisher test.

15.6 Safety end point analysis

The safety end point analysis will include the comparison of adverse reactions (section 15.3), WBC, Hb, PLT and Cr (section 15.5).

16. Handling of missing data

1. The primary outcome analysis should be subject to little or no missing data. All attempts will be made to fully obtain information on 24-week mortality as a minimum for each patient.
2. Missing data that occurs in other outcomes or covariates will be multiply imputed in the primary analysis to increase precision of the estimates and to avoid potential biases from a complete case analysis. Sensitivity analyses will be conducted to assess the impact of the multiple imputation and a complete case analysis will also be conducted. All imputations will be examined to ensure sensible values are being generated. Imputation models will contain baseline variables and outcome variables.
