# Supplementary material for: Mesenchymal Stem Cells Ameliorate Acute‐on‐Chronic Liver Failure Associated With Expansion of Immunoregulatory Hepatic NKp46+DX5− NK Cells
Source: J Immunol Res. 2026 Sep 24;2026:2373331. doi: 10.1155/jimr/2373331 (PMC13602807; doi:10.1155/jimr/2373331)
Supplement: Supplementary file 1 [file JIMR-2026-2373331-s001.zip › Supplementary_File_2.docx]

**Health and Weight Monitoring**

The health status, behavior, and body weight of the mice were closely monitored and documented by the researchers conducting the study.

**Housing Environment and Daily Husbandry ：**Mice were housed in a Specific Pathogen-Free (SPF) animal facility at South China Agricultural University. The daily husbandry and environmental monitoring were strictly managed by certified professional animal care staff. The housing room was maintained under standard controlled conditions, including a 12-hour light/dark cycle, an ambient temperature of 22 ± 2°C, and a relative humidity of 50–60%. Mice were housed in standard individually ventilated cages (IVC) and were allowed unlimited access to standard rodent chow and sterile water.

**During the chronic liver cirrhosis induction phase (Weeks 1-8):** General health and behavior were observed **daily**. Body weights were recorded **twice weekly** prior to each 10% CCl4 administration to adjust the injection volume accurately and assess the animals' tolerance to the modeling process.

**During the acute decompensation phase (ACLF trigger phase):** Following the administration of the sub-lethal dose of 50% CCl4 on day 3 of week 8, the condition of the mice deteriorated rapidly. Consequently, the monitoring frequency was increased. Researchers evaluated the animals' physiological status and behavior every **6 hours** during the 48-hour observation window.

**Humane Endpoints:** To minimize animal suffering during the progressive liver failure model, humane endpoints were strictly predefined and implemented. Researchers were instructed to immediately euthanize (via deep anesthesia followed by cervical dislocation) any animal exhibiting the following criteria before the scheduled experimental endpoints:

1. Rapid or continuous body weight loss exceeding **20%** of the baseline weight.
2. Severe lethargy, prostration, or complete inability to ambulate to reach food and water.
3. Persistent hunched posture, severely ruffled fur, and lack of grooming indicating severe distress.
4. Labored breathing (dyspnea) or loss of the righting reflex.

**Animal monitoring schedule**

| **Date** | **Mouse ID** | **Group** | **Body weight (g)** | **Mental status** | **Activity level** | **Food intake** | **Fur condition** | **Other abnormalities** | **Recorder** |
| --- | --- | --- | --- | --- | --- | --- | --- | --- | --- |
|  |  |  |  | □ Normal  □ Reduced | □ Normal  □ Reduced | □ Normal  □ Reduced | □ Smooth  □ Scruffy |  |  |
