# Supplementary material for: Mesenchymal Stem Cells Ameliorate Acute‐on‐Chronic Liver Failure Associated With Expansion of Immunoregulatory Hepatic NKp46+DX5− NK Cells
Source: J Immunol Res. 2026 Sep 24;2026:2373331. doi: 10.1155/jimr/2373331 (PMC13602807; doi:10.1155/jimr/2373331)
Supplement: Supplementary file 1 [file JIMR-2026-2373331-s001.zip › Supplementary_File_3.pdf]

NOTE: Please save this file locally before filling in the table, DO NOT work on the file within your internet browser as changes will not be saved. Adobe Acrobat Reader (available free here) is recommended for completion.

The ARRIVE guidelines 2.0: author checklist

The ARRIVE Essential 10

These items are the basic minimum to include in a manuscript. Without this information, readers and reviewers cannot assess the reliability of the findings.

Item		Recommendation	Section/line number, or reason for not reporting
**Study design**	1	For each experiment, provide brief details of study design including:<br>a. The groups being compared, including control groups. If no control group has been used, the rationale should be stated.<br>b. The experimental unit (e.g. a single animal, litter, or cage of animals).	Materials and Methods / CCL4 induced acute-on-chronic liver failure murine model: Mice were divided into (1) control, (2) ACLF, (3) ACLF+saline, and (4) ACLF+MSC.<br><br>The experimental unit is a single animal (mouse)
**Sample size**	2	a. Specify the exact number of experimental units allocated to each group, and the total number in each experiment. Also indicate the total number of animals used.<br>b. Explain how the sample size was decided. Provide details of any *a priori* sample size calculation, if done.	Materials and Methods / MSCs Transplantation: n=10 per group for survival assays. Figure legends: n=6 mice/group for flow cytometry, n=12 mice/group for in vitro stimulation.<br><br>Sample sizes were not determined by a priori statistical methods but were chosen based on comparable published studies and prior experience with this specific murine ACLF model, ensuring sufficient statistical power [illegible]
**Inclusion and exclusion criteria**	3	a. Describe any criteria used for including and excluding animals (or experimental units) during the experiment, and data points during the analysis. Specify if these criteria were established *a priori*. If no criteria were set, state this explicitly.<br>b. For each experimental group, report any animals, experimental units or data points not included in the analysis and explain why. If there were no exclusions, state so.<br>c. For each analysis, report the exact value of *n* in each experimental group.	No specific inclusion or exclusion criteria were established a priori<br><br>All experimental animals and collected data points were included in the final analysis; no exclusions were made<br><br>To minimize potential confounders, mice from different experimental groups were co-housed where possible, and cage locations were randomized on the racks. The order of treatments and outcome [illegible]
**Randomisation**	4	a. State whether randomisation was used to allocate experimental units to control and treatment groups. If done, provide the method used to generate the randomisation sequence.<br>b. Describe the strategy used to minimise potential confounders such as the order of treatments and measurements, or animal/cage location. If confounders were not controlled, state this explicitly.	Mice were numbered, and random grouping was performed using a random number table, with equal sample sizes in each of the four groups.<br><br>To minimize potential confounders, mice from different experimental groups were co-housed where possible, and cage locations were randomized on the racks. The order of treatments and outcome assessments (e.g., tissue harvesting) was alternated across groups
**Blinding**	5	Describe who was aware of the group allocation at the different stages of the experiment (during the allocation, the conduct of the experiment, the outcome assessment, and the data analysis).	Due to the nature of the experimental design, investigators were not blinded to group allocation during the conduct of the experiment, outcome assessment, or data analysis
**Outcome measures**	6	a. Clearly define all outcome measures assessed (e.g. cell death, molecular markers, or behavioural changes).<br>b. For hypothesis-testing studies, specify the primary outcome measure, i.e. the outcome measure that was used to determine the sample size.	
**Statistical methods**	7	a. Provide details of the statistical methods used for each analysis, including software used.<br>b. Describe any methods used to assess whether the data met the assumptions of the statistical approach, and what was done if the assumptions were not met.	Statistical Analysis: SPSS 22.0 software was used. Analyses included paired t-tests, Wilcoxon tests, Student's t-tests, Kaplan-Meier with log-rank tests, and Spearman's tests.<br><br>Statistical Analysis: Normality was assessed using Shapiro-Wilk testing.
**Experimental animals**	8	a. Provide species-appropriate details of the animals used, including species, strain and substrain, sex, age or developmental stage, and, if relevant, weight.<br>b. Provide further relevant information on the provenance of animals, health/immune status, genetic modification status, genotype, and any previous procedures.	4- to 6-week-old male Balb/c mice. GFP-transgenic C57BL/6 mice were used for MSC homing tracking.<br><br>Mice were SPF grade
**Experimental procedures**	9	For each experimental group, including controls, describe the procedures in enough detail to allow others to replicate them, including:<br>a. What was done, how it was done and what was used.<br>b. When and how often.<br>c. Where (including detail of any acclimatisation periods).<br>d. Why (provide rationale for procedures).	acute-on-chronic liver failure murine model: 10% CCL4 (5 mL/kg, intraperitoneally) was administered twice weekly for 8 weeks. Acute decompensation was triggered by 50% CCL4 [illegible]<br><br>Twice weekly for 8 weeks, with a final dose on day 3 of week 8, and MSCs injected 1h post-final CCL4 administration.<br><br>Mice were maintained under standard conditions (12h light/dark cycle; allowed unlimited access to food/water).<br><br>To induce liver cirrhosis and a subsequent acute decompensation trigger.
**Results**	10	For each experiment conducted, including independent replications, report:<br>a. Summary/descriptive statistics for each experimental group, with a measure of variability where applicable (e.g. mean and SD, or median and range).<br>b. If applicable, the effect size with a confidence interval.	Data are presented as mean ± SD for continuous variables or frequencies for categorical variables.<br><br>Figure Legends / Figure 1: A 95% confidence interval is provided for the linear regression fit of MELD scores.

The Recommended Set

These items complement the Essential 10 and add important context to the study. Reporting the items in both sets represents best practice.

Item		Recommendation	Section/line number, or reason for not reporting
**Abstract**	11	Provide an accurate summary of the research objectives, animal species, strain and sex, key methods, principal findings, and study conclusions.	Summarizes the therapeutic potential of MSCs
**Background**	12	a. Include sufficient scientific background to understand the rationale and context for the study, and explain the experimental approach.<br>b. Explain how the animal species and model used address the scientific objectives and, where appropriate, the relevance to human biology.	Introduction: Outlines HBV-ACLF pathogenesis, the role of innate immunity, and the lack of understanding of MSC interaction with liver-resident NK cells.<br>Discussion: The CCL4-induced murine ACLF model is widely used to mimic human ACLF conditions and accommodate MSC treatment.
**Objectives**	13	Clearly describe the research question, research objectives and, where appropriate, specific hypotheses being tested.	Introduction: The study aims to characterize M
**Ethical statement**	14	Provide the name of the ethical review committee or equivalent that has approved the use of animals in this study, and any relevant licence or protocol numbers (if applicable). If ethical approval was not sought or granted, provide a justification.	•Animal Ethics: The study was approved by the Animal Ethics Committee of Experimental Animal Ethics Committee of South China Agricultural University (Approval No. 2024h052). The use of human samples was approved by the Ethics Committee of Ethics Committee of The Third Affiliated Hospital of Sun Yat-sen University (Approval No. [2017] No. 17).
**Housing and husbandry**	15	Provide details of housing and husbandry conditions, including any environmental enrichment.	Materials and Methods / CCL4 induced acute-
**Animal care and monitoring**	16	a. Describe any interventions or steps taken in the experimental protocols to reduce pain, suffering and distress.<br>b. Report any expected or unexpected adverse events.<br>c. Describe the humane endpoints established for the study, the signs that were monitored and the frequency of monitoring. If the study did not have humane endpoints, state this.	Results: The ACLF model showed high lethality, with 70% mortality within 48 hours.
**Interpretation/ scientific implications**	17	a. Interpret the results, taking into account the study objectives and hypotheses, current theory and other relevant studies in the literature.<br>b. Comment on the study limitations including potential sources of bias, limitations of the animal model, and imprecision associated with the results.	
**Generalisability/ translation**	18	Comment on whether, and how, the findings of this study are likely to generalise to other species or experimental conditions, including any relevance to human biology (where appropriate).	Discussion: Recommends moving toward HBV-replicating systems or humanized mouse models for improved clinical/translational relevance.
**Protocol registration**	19	Provide a statement indicating whether a protocol (including the research question, key design features, and analysis plan) was prepared before the study, and if and where this protocol was registered.	Acknowledgments: An earlier version of this manuscript was published as a preprint on Research Square. The clinical trial NCT01322906 is also mentioned.
**Data access**	20	Provide a statement describing if and where study data are available.	Data Availability Statement: The data are avail
**Declaration of interests**	21	a. Declare any potential conflicts of interest, including financial and non-financial. If none exist, this should be stated.<br>b. List all funding sources (including grant identifier) and the role of the funder(s) in the design, analysis and reporting of the study.	Disclosure of potential Conflict of Interest: All authors declare no competing financial interests or personal relationships.<br>Funding: Supported by the National Key Research and Development Program of China (2024YFA1108802); National Natural Science Foundation of China (81901940); and Guangdong provincial foundations (2021A1515010306, 2022A1515110769).
