# Supplementary material for: Mesenchymal Stem Cells Ameliorate Acute‐on‐Chronic Liver Failure Associated With Expansion of Immunoregulatory Hepatic NKp46+DX5− NK Cells
Source: J Immunol Res. 2026 Sep 24;2026:2373331. doi: 10.1155/jimr/2373331 (PMC13602807; doi:10.1155/jimr/2373331)
Supplement: Supplementary file 1 [file JIMR-2026-2373331-s001.zip › Supplementary table20260731.doc]

**Supplementary table: Detailed antibody information for multicolour flow cytometry.**

A

| **Target** | **Vendor** | **Clone** | **Cat#** | **Dilution** |
| --- | --- | --- | --- | --- |
| **CD3-V450** | BD | UCHT1 | 560366 | 1:40 |
| **CD56-Percp Cy5.5** | BD | B159 | 560842 | 1:20 |
| **NKP46-PEcy7** | BD | 9E2/NKP46 | 562101 | 1:40 |
| **NKP30-AF488** | R&D | 210845 | FAB1849G | 1:10 |
| **NKG2A-PE** | R&D | 131411 | FAB1059 | 1:40 |
| **NKG2D-APC** | R&D | 149810 | FAB139A | 1:20 |
| **KIR2DL1-Fluorescein** | R&D | 143211 | FAB1844F | 1:10 |
| **KIR2DL3-PE** | R&D | 180701 | FAB2014P | 1:20 |
| **KIR3DL1-APC** | R&D | 177407 | FAB12251A | 1:20 |
| **Perforin-FITC** | eBioscience | Dg9 | 11-9994-41 | 1:10 |
| **Granzyme B-AF700** | BD | GB11 | 560213 | 1:20 |
| **FasL-PE** | Biolegend | NOK-1 | 306407 | 1:20 |
| **Live/dead marker** | Thermo Fisher | None | L10120 | 1:20 |

B

| **Target** | **Vendor** | **Clone** | **Cat#** | **Dilution** |
| --- | --- | --- | --- | --- |
| **CD90-PE** | BD | 553005 | 53-2.1 | 1:40 |
| **CD44-PE** | eBioscience | IM7 | 12-0441 | 1:20 |
| **CD11b-FITC** | eBioscience | M1/70 | 11-0341 | 1:20 |
| **CD73-Percp 710** | eBioscience | TY/11.8 | 46-0731 | 1:10 |
| **MHC-I-FICT** | eBioscience | AF6-88.5.5.3 | 11-5958 | 1:20 |
| **CD106-AF647** | eBioscience | 429 | 50-1061 | 1:20 |

C

| **Target** | **Vendor** | **Clone** | **Cat#** | **Dilution** |
| --- | --- | --- | --- | --- |
| **DX5-PECF594** | BD | DX5 | 562453 | 1:20 |
| **NKP46-Percp-cy5.5** | BD | 29A1.4 | 560800 | 1:40 |
| **CD3-APC-cy7** | biolegend | 17A2 | 100221 | 1:50 |
| **Ly49A-Pacific blue** | biolegend | YE1/48.10.6 | 116809 | 1:20 |
| **LY49C-FITC** | BD | 5E6 | 562055 | 1:10 |
| **NKG2D-APC** | eBioscience | CX5 | 17-5882-81 | 1:50 |
| **NKG2A-PE** | R&D | 705829 | FAB686P | 1:40 |
| **Zombie** | BioLegend | None | 423101 | 1:100 |

D

| **gene symbol** | **Forward** | **Reverse** |
| --- | --- | --- |
| ***Csf2*** | TTTTCCTGGGCATTGTGGTCT | ACTTCTACCTCTTCATTCAACGTG |
| ***Il2*** | TGCGGCATGTTCTGGATTTG | ATGTGTTGTCAGAGCCCTTTAGT |
| ***Ifng*** | GGAGGAACTGGCAAAAGGATG | GTTGCTGATGGCCTGATTGT |
| ***Tnfa*** | CCCACGTCGTAGCAAACCA | TGTCTTTGAGATCCATGCCGT |
| ***Gapdh*** | AGGTCGGTGTGAACGGATTTG | TGTAGACCATGTAGTTGAGGTCA |
